# A divergent haplotype with a large deletion at the berry color locus causes a white-skinned phenotype in grapevine

**DOI:** 10.1093/hr/uhaf069

**Published:** 2025-03-06

**Authors:** Jean-Sébastien Reynard, Justine Brodard, David Roquis, Eric Droz, Komlan Avia, Thibaut Verdenal, Vivian Zufferey, Thierry Lacombe, Daniel Croll, Jean-Laurent Spring

**Affiliations:** Viticulture, Agroscope, Av. de Rochettaz 21, 1009 Pully, Switzerland; Virology, Agroscope, Route de Duillier 60, 1260 Nyon, Switzerland; Hepia, Route de Presinge 150,1254 Jussy, Switzerland; Virology, Agroscope, Route de Duillier 60, 1260 Nyon, Switzerland; INRAE, Université de Strasbourg, UMR SVQV, 68000 Colmar, France; Viticulture, Agroscope, Av. de Rochettaz 21, 1009 Pully, Switzerland; Viticulture, Agroscope, Av. de Rochettaz 21, 1009 Pully, Switzerland; UMR AGAP Institut, CIRAD, INRAE, Institut Agro, Univ Montpellier, F-34398 Montpellier, France; IFV-INRAE-Institut Agro, UMT Geno-Vigne®, F-34398 Montpellier, France; Institute of Biology, Laboratory of Evolutionary Genetics, 2000 Neuchâtel, Switzerland; Viticulture, Agroscope, Av. de Rochettaz 21, 1009 Pully, Switzerland

## Abstract

The current genetic model explaining berry skin color in *Vitis vinifera* is incomplete and fails to predict berry skin color phenotypes for one allele of *VvMybA1,* referred to as *VvMybA1_SUB*. Our study focuses on this specific allele, revealing that the haplotype containing *VvMybA1_SUB* (haplotype F) represents an ancient lineage of the berry color locus. Within haplotype F, we identified two functional subhaplotypes, HapF1 and HapF2, associated with black-skinned phenotype, and one non-functional subhaplotype, HapF^DEL^, responsible for white-skinned phenotype. HapF1 likely originated from wild populations domesticated in the Near East and subsequently spread globally with the expansion of viticulture. In contrast, HapF2 has a more restricted distribution and may have emerged from hybridization events between cultivated grapevines and local wild populations as viticulture migrated to the Italian peninsula. Furthermore, we found that in white-skinned berry cultivar, HapF has undergone a large deletion at the berry color locus, removing the majority of the VvMybA genes. Previous works suggested a single common origin for white-skinned varieties during grapevine domestication. Our results challenge this notion, instead proposing that white-skinned grape cultivars arose at least twice during grapevine domestication history. Alongside the major haplotype A, some white-skinned cultivars, such as cv. ‘Sultanina’ harbor HapF^DEL^. Since HapF^DEL^ is present only in table grape varieties, we suggest that it likely arose from a recent mutational event and dispersed along the ancient Silk Road into East Asia. These findings enhance our understanding of the genetic diversity and evolutionary trajectory of grapevine cultivars, offering insights into their domestication and spread across different geographical regions.

## Introduction

Since the seminal work of Gregor Mendel (1822–1884) in the middle of the 19th century, the inheritance and genetic bases of color variation in plants have attracted significant scientific interest. The red, purple, and blue coloration of plant tissues is mainly caused by a class of pigments called anthocyanins. These secondary metabolites are stored in plant vacuoles and belong to the flavonoid class. Their biosynthesis is a branch of the phenylpropanoid pathway, originating from phenylalanine [1]. Anthocyanins are crucial for various plant physiological processes and play a major role in ecological interactions, including those with pollinators and seed dispersers [2]. In Eurasian grapevine (*Vitis vinifera*), anthocyanins begin to accumulate in berry skin at veraison (i.e., the onset of ripening). This accumulation is influenced by both genetic factors and the environment. The presence or absence of anthocyanins in berry skin is crucial for producing wines of various colors. Additionally, berry color is a key criterion for ampelography (i.e., the description of the different grapevine cultivars), allowing the classification of thousands of different cultivars into two primary categories based on whether anthocyanins are present in the berry skin.

Most of the cultivars grown today are the result of a domestication process from the wild ancestor *Vitis vinifera* subsp. *sylvestris* (hereafter *V. sylvestris*). Since wild grapes are always blue/black at maturity, a trait associated with attracting birds for seed dispersal, the original form of *V. sylvestris* most likely exhibited a black-skinned phenotype [3]. Evidence suggests that white wine was already produced in Egypt during the reign of pharaoh Tutankhamun (1341-1323BCE) [4], although almost all the documents attest to a cultivation of black grapes. White cultivars were already known to be cultivated by ancient civilizations during antiquity, such as the Greeks and Romans.

**Figure 1:**
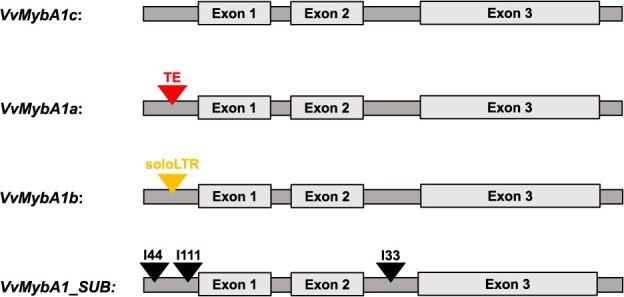
Schematic illustration of different alleles of the *VvMybA1* gene. *VvMybA1c* is the functional allele; *VvMybA1a* is the non-functional white allele containing *Gret1*, a transposable element (TE), in the promoter region of the gene. For *VvMybA1b*, *Gret1* is partially excised, and only a portion (soloLTR) remains in the promotor region. The *VvMybA1_SUB* allele has three small insertions (black triangles: 44 bp, 111 bp, and 33 bp) compared to *VvMybA1c*.

White grape varieties do not produce anthocyanins in berry skin during the ripening phase. Classic genetic experiments have long shown that for *V. vinifera*, berry skin color is controlled by a major locus, and the white-skinned berry allele is recessive [5]. The first work to identify and characterize some genes from the flavonoid pathway in *V. vinifera* was carried out 30 years ago [6]. Shortly after, others have demonstrated that unlike in grape varieties of other colors, *VvUFGT* (UDP-glucose: flavonoid-3-O-glucosyltransferase) expression is absent in white-skinned cultivars [7]. The enzyme UFGT catalyzes the glycosylation of unstable colorless anthocyanidin aglycones into pigmented anthocyanins. *VvUFGT* was later shown to be transcriptionally regulated by *MYB* factors [8]. The first *MYB* gene was initially identified as an oncogene in an avian myeloblastosis virus [9], and similar genes were found later in plants. Numerous studies have indicated that the *MYB* family constitutes one of the largest transcription factors in plants, and some *MYB* factors have been shown to be involved in the anthocyanin biosynthesis pathway in *Zea mays* [10]. In *V. vinifera*, a region on chromosome 2 has been identified as the major genetic determinant of berry skin coloration and is called the berry color locus (BCL) [11]. This locus was shown to contain multiple copies of *MYB* genes, called *VvMybA* followed by a number, that control berry coloration through *VvUFGT* transcriptional regulation [12, 13]. These previous studies showed that in colored berry cultivars, two paralogs (*VvMybA1* and *VvMybA2*) located at the BCL are functional. Given that *VvMybA1* and *VvMybA2* are two neighboring genes, they are largely inherited together and can be considered a block or haplotype. The haplotype C-N containing both functional *VvMybA1c* and *VvMybA2r* is able to activate *VvUFGT* expression and to produce berry skin pigmentation [14].

In white-skinned berry cultivars, the two functional genes, *VvMybA1* and *VvMybA2,* are mutated and constitute haplotype A (HapA). In the case of *VvMybA1* from HapA (*VvMybA1_HapA* or *VvMybA1a*), gene expression is repressed as a consequence of the insertion of the retrotransposon *Gret1* into the promoter region [15]. For *VvMybA2_HapA,* two nonsynonymous mutations inactivate the protein [12]. HapA is the major haplotype responsible for white-skinned berry cultivars, as it is homozygous in almost all white grape varieties [12]. However, there are a few exceptions. First, in the case of clonal variation, some white-skinned clones originate from black-skinned cultivars through mutations (e.g. *‘*Pinot noir’ > ‘Pinot blanc’). In these cases, the underlying molecular mechanism is fully understood and involves a large deletion at the BCL [16–18]. Second, Lijavetsky *et al.* described the allele *VvMybA1_SUB* in ‘Sultanina’ and reported four white-skinned berry cultivars not homozygous for HapA [19]. The *VvMybA1_SUB* allele might belong to an independent putative haplotype, and was named haplotype F (HapF) [20]. Knowledge of HapF is limited, and several aspects remain unknown. For example, what about other *VvMybA* genes that might be present at the BCL for this haplotype?

In this study, we address these questions through a combination of PCR assays, Sanger sequencing, Oxford nanopore long-read sequencing (ONT), *de novo* assembly, whole genome sequencing (WGS), and transcriptomic approaches. We analyzed data from more than 500 cultivars originating from diverse countries around the world. We then reconstructed BCL for two cases: (i) HapF in a black-skinned cultivar that originated from the Alps and (ii) HapF from a white-skinned cultivar ‘Khusaine Belyi’. We found that HapF in the white-skinned berry cultivar has undergone a large deletion that removed the majority of *VvMybA* genes at the BCL. In summary, HapF could be subdivided into three subhaplotypes—a non-functional HapF^DEL^ and two functional subhaplotypes, HapF1 and HapF2—with contrasting evolutionary histories. Finally, our results showed at least two origins for white grape cultivars in *V. vinifera*, contrary to expectations.

## Results

### The *VvMybA1_SUB* allele is present in colored-skinned and white-skinned berry cultivars

To estimate the geographical distribution and frequency of the *VvMybA1_SUB* allele, 528 *V. vinifera* accessions selected to represent the different origins from our grapevine collections were analyzed. Four different *VvMybA1* alleles were observed (Fig. 1). We identified 71 different accessions carrying the *VvMybA1_SUB* allele (Table S1). The *VvMybA1_SUB* allele frequency was 7%, lower compared to other alleles (e.g., 66% for the non-functional white allele *VvMybA1a* and 27% for the functional allele *VvMybA1c)*. One cultivar was of particular interest: two alleles, *VvMybA1_SUB* and *VvMybA1a*, were detected in leaves of ‘Humagne gris’, whereas only the white allele *VvMybA1a* was detected in root extracts.

**Figure 2:**
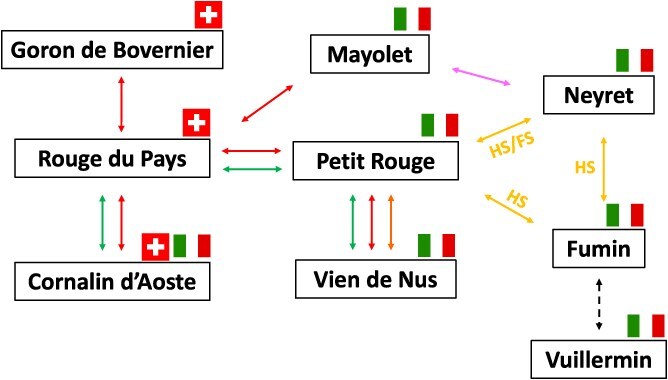
Genetic relationships between nine black-skinned cultivars from the Alps region according to the literature (if not specified, each arrow corresponds to a parent/offspring relationship according to different sources: Raimondi *et al.* [22] in yellow, Onofrio *et al.* [23] in green, Lacombe *et al.* [24] in red, Cipriani *et al.* [25] in violet and the dashed line indicates that at least one allele is shared across 9 SSR markers, as referenced on www.vivc.de). All nine cultivars carry the *VvMybA1_SUB* allele. HS and FS stand for half or full sibling. Flags indicate where each cultivar is grown: Switzerland (Valais) and/or Italy (Aosta Valley).

The *VvMybA1_SUB* allele was observed in cultivars originating from diverse countries. We did not find any French or German cultivars carrying *VvMybA1_SUB*. Only two Spanish cultivars (‘Listan Prieto’ and ‘Mollar Cano’) and one cultivar from Portugal (‘Tinta Francisca’) were shown to carry *VvMybA1_SUB*. By contrast, *VvMybA1_SUB* was identified in several cultivars from Italy (Table S1). Furthermore, this allele was identified in nine black-skinned cultivars from the Alps region (i.e. ‘Mayolet’, ‘Petit Rouge’, ‘Rouge du Pays’, ‘Goron de Bovernier’, ‘Cornalin d’Aoste’, ‘Neyret’, ‘Fumin’, ‘Vuillermin’, and ‘Vien de Nus’). Some of these genotypes are cultivated on both sides of the Swiss–Italian border (Valais and Aosta Valley). The nine alpine cultivars belong to a group of genetically closely related genotypes as illustrated by the IBD estimates (Fig. 2, Table S2). By comparing the proposed pedigrees of these cultivars with allelic composition data at *VvMybA1*, we identified a discrepancy. The cultivar ‘Rouge du Pays’ (syn. ‘Cornalin du Valais’) was found to have a *VvMybA1_SUB / VvMybA1c* genotype despite the suggested parents cv. ‘Mayolet’ and ‘Petit Rouge’ [21], both having a *VvMybA1_SUB / VvMybA1a* genotype. The plant material was genotyped with nine genome-wide SSR markers to verify the identity for trueness-to-type of cv. ‘Mayolet’, ‘Petit Rouge’, and ‘Rouge du Pays’ (http://www.vivc.de/). To further investigate the pedigree, nine additional SSR markers were analyzed, among which four markers were incompatible with the pedigree suggested by Vouillamoz *et al.* [21] for the cultivar ‘Rouge du Pays’ (Fig. S1).

Combining our analyses with published data (Table S3), we found that among 62 cultivars sharing genotype *VvMybA1_SUB / VvMybA1a*, 16 accessions had white-skinned berries, eight had light-colored-skinned berries (i.e. red or rose), and 38 had black-skinned berries. Comparative sequence analysis of the *VvMybA1_SUB* allele showed that the color difference could not be explained by variation in coding sequences of gene *VvMybA1*, given that they were identical. The co-occurrence of colored and white-fruited phenotypes among cultivars with genotype *VvMybA1_SUB / VvMybA1a* might indicate additional regulatory loci controlling berry skin color.

### The berry color locus of haplotype F in alpine black-skinned berry cultivars

**Figure 3:**
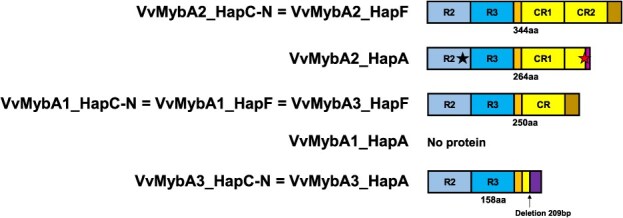
Representation of the predicted MybA proteins produced from MYB genes at the color locus on chromosome 2, depending on the haplotype. Colored boxes are used to denote similar regions. R2 and R3 refer to repeats in c-Myb. The C-terminal domain (CR) is repeated in *VvMybA2*_*HapC-N* and *VvMybA2_HapF*. The black star in *VvMybA2*_*HapA* indicates a non-conservative change (R^44^L), and the red star stands for a dinucleotide deletion introducing a premature stop codon. The protein length is given in amino acids (aa). Haplotype A produces no *VvMybA1* protein, given the insertion of a transposable element in the promoter region of the gene. *VvMybA3* in haplotypes C-N and A lacks most of the C-terminal domain, due to a 209 bp deletion in the coding region of the gene. *VvMybA2* and *VvMybA3* encode proteins with a similar structure in both haplotypes F1 and F2. *VvMybA1_HapF* is present in all three subhaplotypes (F1, F2, and F^DEL^; see Table 1) and also exhibits a similar structure.

The BCL region was further analyzed in a homozygous genotype (*VvMybA1_SUB/VvMybA1_SUB)* derived from an alpine black-skinned cultivar. First, using PCR analysis, three *MybA*-related sequences, including the complete coding region, were obtained for the following genes: *VvMybA1* (PQ072838, 1820 bp), *VvMybA2* (PQ072839, 2361 bp), and *VvMybA3* (PQ072840, 2072 bp). Given that *VvMybA1*, *VvMybA2*, and *VvMybA3* are adjacent genes, they are inherited together and can be considered haplotypes. The different haplotypes described for the BCL are summarized in Table 1, along with their allelic composition. The analysis of the coding sequences and protein products of the three genes from HapF are presented in Fig. 3. We found that (i) *VvMybA1_SUB* (= *VvMybA1_HapF*) encoded a protein of 250 amino acids in length; (ii) *VvMybA2_HapF* had a similar structure and length as *VvMybA2* from haplotype C-N: the duplication of a 282 bp-portion of the C-terminal domain in *VvMybA2_HapF* is of the same size as *VvMybA2r* from haplotype C-N [12]. Further, *VvMybA2_HapF* did not show the CA deletion and the mutation R- > L at position 44 of the protein described for the white allele *VvMybA2w* [12]; and (iii) contrary to what has been described for *VvMybA3* in other haplotypes (C-N, A), *VvMybA3_HapF* encoded a protein of similar length to *VvMybA1* due to the absence of the deletion of 209 bp observed in the third exon [12].

**Table 1:** Summary of the different haplotypes at the BCL. Allelic composition is given for every haplotype together with an example of a cultivar bearing it. *Gret1* is a retrotransposon

**Haplotype**	**Species**	**Example of acultivar**	***VvMybA1***	***VvMybA2***	***VvMybA3***	***Berry skin color***	***Comments***
HapA	*Vitis vinifera*	‘Chasselas’	VvMybA1a: *Gret1* insertion in the promoter region, nonfunctional	2 nonsynonymous mutations, nonfunctional	209 bp deletion in the third exon, nonfunctional	Not able to trigger anthocyanin production	
HapB	*Vitis vinifera*	‘Chardonnay rose’	VvMybA1b: *Gret1* is partially excised	2 nonsynonymous mutations, nonfunctional	209 bp deletion in the third exon, nonfunctional	Partially able to trigger anthocyanin production	
HapC-N	*Vitis vinifera*	‘Pinot noir’	VvMybA1c: functional	VvMybA2r: functional	209 bp deletion in the third exon, nonfunctional	Trigger anthocyanin production	
HapD	*Vitis vinifera*	‘Pinot blanc’	Absent, large deletion at the BCL	Absent, large deletion at the BCL	Absent, large deletion at the BCL	Not able to trigger anthocyanin production	
HapE1	*Vitis labrusca*	‘Concord’	Absent, large deletion at the BCL	VlMybA1-2	VlMybA1-3	Trigger anthocyanin production	
HapF1	*Vitis vinifera*	‘Saperavi’	VvMybA1_SUB, likely functional	Likely functional	No 209 bp deletion, likely functional	Trigger anthocyanin production	No tandem duplication of *VvMybA4*
HapF2	*Vitis vinifera*	‘Montepulciano’	VvMybA1_SUB, likely functional	Likely functional	No 209 bp deletion, likely functional	Trigger anthocyanin production	Tandem duplication of *VvMybA4*
HapF^Del^	*Vitis vinifera*	‘Sultanina’	VvMybA1_SUB, likely functional	Absent, large deletion at the BCL	Absent, large deletion at the BCL	Not able to trigger anthocyanin production	

**Figure 4:**
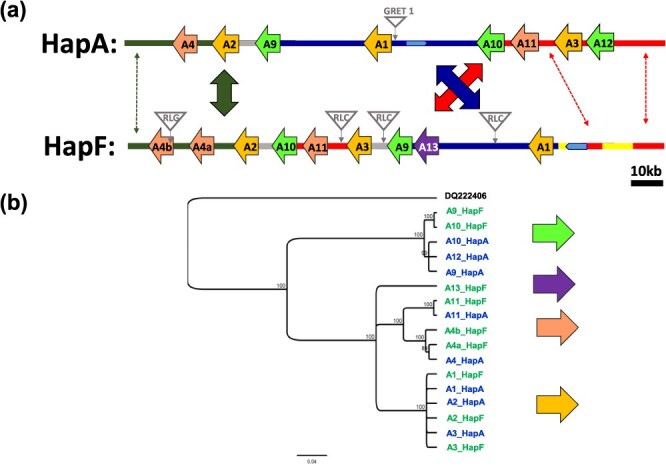
Organization of the berry color locus (BCL) on chromosome 2 for two haplotypes present in *Vitis vinifera*. HapA is the canonical non-functional haplotype from PN40024, while haplotype F is the one containing *VvMybA1_SUB*. **a** Schematic representation of the cluster of MybA genes (denoted here by A followed by a number) found in both haplotypes. Similar colors denote regions with similarities. The grapevine retrotransposon (Gret1) is shown in the *VvMybA1* promoter region of haplotype A. Transposable element insertions specific to haplotype F are shown as gray triangles, and two superfamilies were identified: Copia (RLC) and Gypsy (RLG); **b** Phylogenetic tree constructed from the different nucleotide sequences of *MybA* genes identified at BCL for both haplotypes A and F. The sequences are colored according to the haplotypes in which they have been identified (HapA in blue and HapF in green). *MybA75* (*DQ222406*) from *Arabidopsis thaliana* is used as the outgroup. The scale bar shows the number of substitutions per site.

Second, the homozygous genotype was analyzed using ONT long-read genome sequencing. This sequencing experiment was conducted using a complete flow cell and generated 4.7 million reads (N50: 10.4 kb, passed bases: 22.5 Gb). After a long read de novo assembly, one contig (length: 3.8 Mbp) was identified to correspond to a part of chromosome 2 containing the color berry locus (Fig. S2). At this locus, nine different *VvMybA*-like sequences were identified over a region of approximately 138 kb. The sequences of those nine *VvMybA* genes were polished and corrected manually at the base-pair level using Illumina short read to correct long read sequencing errors. A comparison between the three sequences obtained by Sanger sequencing of PCR amplicons and genome assembly indicates a 100% identity over 6253 bp between the two approaches. Both berry color haplotypes (HapF vs. HapA in PN40024.v4) were compared using a dotplot (Fig. S3), and the inferred genomic organization from HapF is depicted in Fig. 4. The *MYB* sequence cluster organization for HapF is different from what has been reported for HapA. First, the order of the *MYB* sequences is not conserved between both haplotypes (Fig. 4, Fig. S3). Second, the number of *MYB* sequences in the cluster is not identical between haplotypes (8x for HapA or HapC-N, 9x for HapF). Third, at the BCL of HapF, we observed a tandem duplication (6.1 kb, 6.4 kb, 90% nucleotide identity) in the region containing *VvMybA4* (Fig. S3), resulting in two gene copies, denoted *VvMybA4a* and *VvMybA4b*. Finally, one *MYB* sequence from HapF, *VvMybA13*, has no close homolog in HapA (Fig. 4) and is distantly related to the VvMybA1-2-3 and VvMybA4-11 groups (nucleotide identity: 60–78%).

HapF was analyzed for transposable elements (TE). Four complete retrotransposons (with tandem site duplication and long terminal repeat (LTR) on both sides) were found in HapF that are not present in HapA (Fig. 4). The insertion times of the four intact LTR retrotransposons were estimated by comparing their LTR nucleotide divergences (Table S4). Insertion age estimates for these TE ranged from 0.19 to 1.19 million years ago. For comparison, using the same method, the insertion time of the retroelement *Gret1* described in HapA and partially responsible for the white-skinned berry phenotypes was estimated to be 0.15 million years ago.

### Identification of a large deletion at the berry color locus of white-skinned cultivars bearing allele *VvMybA1_SUB*

**Figure 5:**
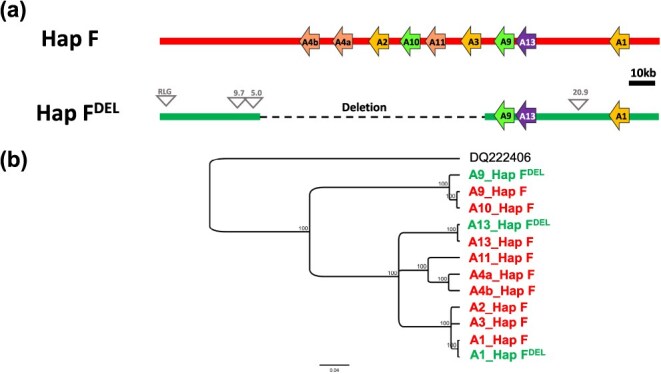
Organization of the berry color locus (BCL) for haplotype F compared to F^DEL^. **a** Schematic representation of the cluster of *MybA* genes (denoted here by A followed by a number) fount at the BCL. The deleted region in HapF^DEL^ compared to HapF is indicated with a dashed line. Insertions specific to haplotype F^DEL^ are shown as gray triangles (RLG for TE from Gypsy superfamily, for other insertions, size (in kb) is written above each insertion). **b** Phylogenetic tree constructed from the different nucleotide sequences of *MybA* genes identified at the BCL from both haplotypes F and F^DEL^. The sequences are colored according to the haplotype in which they have been identified (haplotype F in red and haplotype F^DEL^ in green). *MybA75* (*DQ222406*) from *Arabidopsis thaliana* is used as the outgroup. The scale bar shows the number of substitutions per site.

To determine the portion of chromosome 2 around the *VvMybA1_SUB* gene in a white-skinned berry cultivar, cv. ‘Khusaine Belyi’ was selfed to obtain a homozygous genotype (*VvMybA1_SUB / VvMybA1_SUB*). We then used ONT long-read sequencing with an entire flow cell producing 5.9 M reads (N50: 10.6 kb, passed bases: 30.7 Gb) for the *de novo* assembly. One contig (length: 3.9 Mbp) corresponded to part of chromosome 2 (Fig. S4), including the BCL. A comparison of the BCL of HapF with the newly assembled BCL sequence revealed a large deletion of approximately 76 kb (Fig. S5). Therefore, this haplotype containing the partial deletion was named HapF^DEL^. The genomic organization of the BCL for this haplotype is depicted in Fig. 5. Among the nine *MYB* sequences identified at the BCL previously for HapF, only three are present in HapF^DEL^, namely *VvMybA1*, *VvMybA9*, and *VvMybA13*. The three sequences were highly similar to HapF (pairwise nucleotide identity: 98.6% to 99.3%; Fig. 5). The deleted segment mapped to 1.4 kb upstream of the gene *VvMybA3* and extended 10.8 kb downstream of the gene *VvMyb4b*. Thus, the region containing the 6 *MYB* sequences (*VvMybA3, VvMybA11, VvMybA10, VvMybA2, VvMybA4a*, *and VvMybA4b*) was absent from HapF^DEL^ compared to HapF.

To differentiate HapF from HapF^DEL^, we developed a PCR assay that specifically targeted *VvMybA3* from HapF using specific INDELs (Fig. S6). The assay confirmed the absence of *VvMybA3_HapF* in genotypes HapA/HapA and HapC-N/HapA (data not shown). We used this assay across several accessions found to carry *VvMybA1_SUB* (Table S1). Among the 51 accessions with genotype *VvMybA1_SUB*/*VvMybA1_HapA*, 33 accessions produced positive outcomes in the assay, indicating the presence of the gene *VvMybA3_HapF*, and all these accessions presented a black-fruited phenotype. Among the 18 accessions lacking *VvMybA3_HapF*, we found 16 genotypes producing white-skinned berries and 5 light-colored (red or rose) berries. No black-skinned berries were produced from genotypes lacking *VvMybA3_HapF* (Table S1). Due to the very similar sequences between the different *VvMybA2* alleles, we were unable to design a PCR assay to specifically distinguish the gene *VvMybA2_HapF*. Nevertheless, we examined the whole genomic sequencing data of seven accessions harboring HapF^DEL^ from various geographical origins (Table S8). The HapF^DEL^ appeared similar in the seven accessions, showing the same deletion pattern. HapF^DEL^ was not found in western European cultivars used for wine making but was present in cultivars originating from Asia (mainly Caucasia and central Asia) used as table grapes (Table S1).

### Allele *VvMybA1_SUB* is expressed at a low level in both white- and black-skinned cultivars

To study *MybA* gene expression in the skin after veraison, RNA-Seq transcriptome analysis was performed on four cultivars, two black-skinned (cv. ‘Cornalin d’Aoste’ and ‘Listan Prieto’) with genotype HapF/HapA at the BCL and two white-skinned cultivars (cv. ‘Khusaine Belyi’ and ‘Otcha Bala’) with genotype HapF^DEL^/HapA. In those transcriptomes, based on RNA read alignments, no evidence was found for sequences *VvMybA4a*, *VvMybA4b*, *VvMybA9*, *VvMybA10*, *VvMybA11*, and *VvMybA13* of HapF. Six different cDNAs (*VvMybA1_HapF*, *VvMybA2_HapF*, *VvMybA3_HapF*, *VvMybA1_HapA*, *VvMybA2_HapA*, and *VvMybA3_HapA*) were aligned. We identified private SNPs present in only one *VvMybA* allele (Table S5). We then estimated the proportion of each *MybA*-type sequence in the four transcriptomic datasets, averaging the allele frequencies of the corresponding private SNPs (Table S6). Using this information, we estimated the transcript per million metrics (TPM) for each gene/allele as a measure of gene expression (Table 2). As expected from the genomic analysis, neither *VvMybA2_HapF* nor *VvMybA3_HapF* transcription was detected in the white-skinned cultivars. By contrast, *VvMybA1_HapF* was detected in all four transcriptomic datasets, although at a lower rate compared to other expressed *MybA* genes. As anticipated, UFGT expression differed significantly between white- and black-fruited cultivars, emerging as one of the genes with the highest fold-change when comparing gene expression between the two groups (data not shown).

**Table 2:** Gene expression data (TPM, transcripts per million) in berry skin at six weeks after veraison for four cultivars. BCL stands for berry color locus on chromosome 2

**Cultivar**	**Genotype at BCL**	**Skin berry color**	**Gene/allele expression (TPM)**						
			**Haplotype F/F** ^**DEL**^			**Haplotype A**			
			***VvMybA1***	***VvMybA2***	***VvMybA3***	***VvMybA1***	***VvMybA2***	***VvMybA3***	***UFGT***
‘Cornalin d’Aoste’	HapF/HapA	Black	33	98	104	n. d.	81	218	141
‘Listan Prieto’	HapF/HapA	Black	2	23	95	n. d.	35	70	149
‘Otcha Bala’	HapF^DEL^/HapA	White	9	n. d.	n. d.	n. d.	18	80	0.4
‘Khusaine Belyi’	HapF^DEL^/HapA	White	5	n. d.	n. d.	n. d.	24	85	0.4

We also examined a transcriptomic dataset generated from cv. ‘Sultanina’ berry skin tissues after veraison [26]. No expression of *VvMybA1_SUB* was identified in this dataset (Table S6). To further analyze gene expression, we used two RT-PCR assays combined with Sanger sequencing. First, using primers specific to *VvMybA1* (vvMybA1_f / vvMybA1_r), we specifically detected *VvMybA1_HapF* expression based on one SNP present in *VvMybA1_HapF* (Fig. S7). *VvMybA1_HapF* expression was observed in three white-skinned cultivars—‘Khusaine Belyi’, ‘Otcha Bala’, and ‘LN33’—and two black-fruited cultivars—‘Listan Prieto’ and ‘Cornalin d’Aoste’. However, the private SNP for *VvMybA1_HapF* expression was not detected in cv. ‘Sultanina’ (Fig. S7). Second, we developed another RT-PCR assay (vvMybA1_f / vvMybA1_Ex3_r) that produced a longer amplicon (530 bp) and specifically amplified only two cDNAs: *VvMybA1_HapF* and *VvMybA3_HapF*. Along the amplified region, 12 SNPs differentiated the two cDNAs. An inspection of the sequencing chromatograms at these 12 positions revealed double picks (Fig. S8). For both cv. ‘Cornalin d’Aoste’ and ‘Listan Prieto’, each peak related to the private SNP of *VvMybA1_HapF* had a systematic lower intensity compared with the peak produced by allele *VvMybA3_HapF*.

### Haplotype F is present in *V. vinifera* subsp. *sylvestris*

To investigate the evolution of *MybA* sequences, we examined the homologous region of BCL on chromosome 2 of four accessions of *V. sylvestris* [27] (www.grapegenomics.com). All four genotypes showed a *VvMybA2* allele containing a duplicated portion (282 bp) of the C-terminal domain. Additionally, we identified two *V. sylvestris* accessions, DVIT3351.27 and O34-16, showing a very similar structure at the BCL as HapF described in this work (Fig. S9). In fact, eight *MybA* sequences were identified in those two *V. sylvestris* accessions that were highly similar (>99% nucleotide identity) to those from HapF described here. No evidence of segmental duplication was detected in the *VvMybA4* region for both *V. sylvestris* accessions. Based on the different features observed for genes *VvMybA4a* and *VvMybA4b,* we propose an evolutionary scenario for this locus (Fig. S10). The first step was a tandem duplication, and using the same method for dating TE insertion, this tandem duplication was estimated to have occurred 1.2 million years ago. In the second phase, a TE inserted in the first exon of one copy of *VvMybA4* and a 213-bp insertion occurred in the third exon of the other copy of *VvMybA4.* The age of this TE insertion was estimated at 0.69 million years ago by comparing the LTRs (Table S4) older than the *Gret1* insertion. We called HapF2 the haplotype that contained the tandem duplicated *VvMybA4* and F1 the other haplotype.

To estimate the distribution, frequency, and type of haplotype F in *V. sylvestris,* we obtained archived sequence reads of WGS for 189 accessions (Table S7). In the WGS data for V. *sylvestris*, HapF2 was absent, and 40 accessions bearing HapF1 were detected (Table 3). HapF1 was absent in all tested accessions from Europe. By contrast, going east, in regions such as the Fertile Crescent (near the Mediterranean) and Caucasia, HapF1 was frequent in the *V. sylvestris* population. Furthermore, to estimate the distribution and type of HapF in *V. vinifera* subsp*. sativa*, we resequenced 5 alpine cultivars and analyzed 28 archived WGS for cultivars bearing *VvMybA1_SUB* (Table S8). Out of 33 cultivars, 7 had HapF^DEL^; among the remaining 26 cultivars, HapF2 (containing the tandem duplication) was observed only in alpine cultivars and in cv. ‘Montepulciano’ (two independent WGS datasets). In all 17 other tested cultivars with diverse origins (i.e. from Spain to Uzbekistan), we found HapF1. Finally, we analyzed grape genome sequencing data related to archeological samples [28] and found that one cultivar, A33, carried HapF1 (Table S8).

**Table 3:** Haplotype F1 frequency detected in whole genomic sequencing (WGS) data in diverse accessions of *Vitis vinifera* subsp. *sylvestris* from different origin. The details of the studied WGS data are given in Table S7

**Region/country where the accessions were collected**	**Number of WGS data examined**	**HapF1 detected (frequency in %)**
Portugal	4	Not detected
Spain	20	Not detected
Tunisia	2	Not detected
France	10	Not detected
Europe	9	Not detected
Switzerland	5	Not detected
Germany	5	Not detected
Austria	4	Not detected
Italy	40	Not detected
Croatia	5	Not detected
Hungary	5	Not detected
Bulgaria	5	Not detected
Roumania	3	Not detected
Turkey	5	Not detected
Crimea	10	1 (10%)
Israel	11	7 (64%)
Iran	10	6 (60%)
Caucasia	6	3 (50%)
Armenia	10	8 (80%)
Azerbaijan	10	6 (60%)
Georgia	10	9 (90%)

### Analysis of *MybA* genes at the berry color locus in *Muscadinia rotundifolia* and *Vitis labrusca*

**Figure 6:**
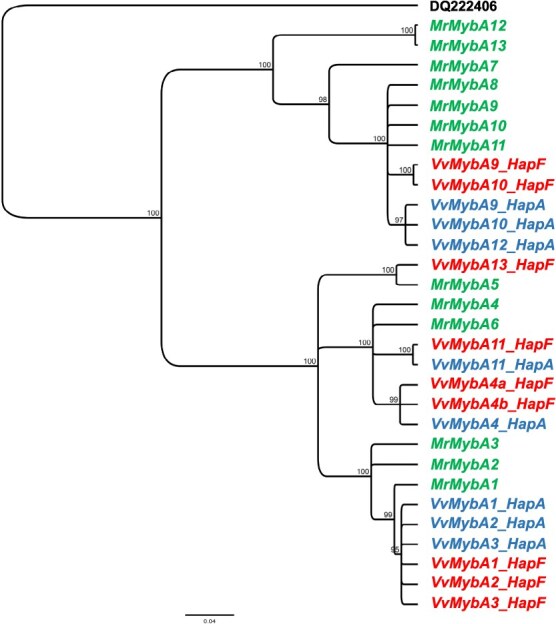
Phylogenetic tree constructed from different nucleotide sequences of *MybA* genes identified at BCL for *Vitis vinifera* and *Muscadinia rotundifolia* (H2) (Cochetel et al. [29]). The sequences are colored according to the haplotypes in which they have been identified. Haplotype A in blue and haplotype F in red were identified in *Vitis vinifera*, whereas haplotype H2 in green originated from *Muscadinia rotundifolia*. *MybA75* (*DQ222406*) from *Arabidopsis thaliana* is used as the outgroup. The scale bar shows the number of substitutions per site.

We examined the homologous region of the BCL on chromosome 2 in *Muscadinia rotundifolia*. We used the data of chromosome-scale pseudomolecules published by Cochetel *et al.* [29] for the cultivar ‘Trayshed’. We compared haplotype H2 with chromosome 2 of PN40024. The two sequences were syntenic, except for a large inversion containing the BCL (Fig. S11). Haplotype 2 in *Muscadinia rotundifolia cv. ‘*Trayshed’ contains three *MYB* sequences similar to genes *VvMybA1* from *V. vinifera* (Fig. 6). Two of these sequences could represent functional genes and encode proteins of size 250 aa (MrMybA1) or 247 aa (MrMybA2) with high similarity to *VvMybA1* (identity with DQ886417: 92–97%). A third sequence (*MrMybA3*) represents a likely pseudogene, since a TE belonging to the *Copia* family has been inserted in the third exon. Regarding these three *MybA1*-like sequences*,* we observed that all had a second intron very similar to *VvMybA1_SUB* containing an insertion of 33 or 35 bp compared with the reference allele *VvMybA1c* (Fig. S12). Furthermore, the sequence *VvMybA13* from HapF in *V. vinifera* had a close homologous sequence in both haplotypes from *Muscadinia rotundifolia* cv. Trayshed (length: 1.2 kb; nucleotide identity: 92%) (Fig. 6). We made similar observations from the genomic data for another variety, *Muscadinia rotundifolia cv. ‘*Noble’ [30]: (i) the presence of three *MybA1*-like sequences, (ii) a second intron containing an insertion very similar to *VvMybA1_SUB*, and (iii) the presence of a homologous sequence of *VvMybA13_HapF.*

 Finally, we examined the homologous region of the BCL on chromosome 2 of one individual of the species *V. labrusca*. We used the data published by Li and Gschwend (2023) for the genotype Grem-4. We compared the BCL region of Grem-4 with PN40024. Both regions had a high collinearity (Fig. S13). Seven *MYB* sequences were identified at the BCL of Grem-4 instead of eight for PN40024. This was explained by a large deletion (approximately 89 kb) that contained *VvMybA1* (Fig. S13). *VlMybA1-2* and *VlMybA1-3*, two *MYB* sequences reported previously in *V. labrusca* [20], were 100% identical to the BCL region in Grem-4. As shown by the dotplot analysis (Fig. S13), we suggest that *VlMybA1-2* and *VvMybA2* are orthologs; the same applies for the pair *VlMybA1-3* and *VvMybA3*. Contrary to what has been described in *V. vinifera* for *VvMybA2,* gene *VlMybA1-2* did not show the 282-bp duplicated segment but a 9-bp deletion in the third exon compared to *VvMybA2.*

### QTL analysis of wine anthocyanin content

‘Artaban’ and ‘Divico’ are two new grape varieties with resistance to fungal pathogens. Both are black-skinned berry cultivars heterozygous at the BCL. Indeed, out of 187 genotypes from a cross between those two cultivars, 36 progenies were white-skinned and 151 were black-skinned berry cultivars. These observations are consistent with a genetic model consisting of a single locus and black fruited being dominant over white fruited phenotype with a segregation ratio of 3:1 (chi-squared value = 3.29, *p* = 0.07). We made wines over three seasons and measured their anthocyanin content. The genotype effect determining wine anthocyanin content was evident, given the correlation between trait values across years and among genotypes (Fig. S14). QTL analysis for wine anthocyanin content showed a major QTL on chromosome 2 in the region of the BCL. Haplotype composition at this position had an important effect on wine anthocyanin content. The black-fruited progenies were divided into homozygous at the BCL with two copies of a functional haplotype, and heterozygous at BCL with a functional and a non-functional haplotype (i.e., HapA) (Fig. S15). The mean anthocyanin content was significantly higher (+71% on average over three years, *p* = 3 × 10^−13^) in wines made from genotypes having two functional haplotypes compared to the ones having only one.

## Discussion

In this study, we conducted a deep analysis of the diversity in *VvMybA1* alleles present in grapevine germplasm collections. Similar to other studies [19, 31, 32], our results showed that *VvMybA1_SUB* is a minor allele that is less frequently found than other alleles such as *VvMybA1a* and *VvMybA1c*. We observed this allele in various cultivars from Western Europe. For instance, ‘Listan Prieto’, an ancient Spanish cultivar, was found to carry allele *VvMybA1_SUB. ‘*Listan Prieto’ has also been cultivated for centuries in the Americas, where it is known by different synonyms, including ‘País’, ‘Criolla chica’, ‘Negra Corriente’, and ‘Mission’ [33, 34]. We also detected the *VvMybA1_SUB* allele in progenies of ‘Listan Prieto’ from South America (i.e. Criollas cultivars, e.g. ‘Quebranta’ and ‘Cereza’).

Valais in Switzerland and the Aosta Valley in Italy are neighboring regions in the Alps with a rich winegrowing heritage that dates back to at least the Roman era. During Roman times, a road was constructed over Mons Jovis, now known as the Great St Bernard Pass, which connected these two valleys. During the Middle Ages, pilgrims walked along *Via Francigena*, and this axis represented an important route between northern and southern Europe. On both sides of the border, indigenous grape cultivars have been cultivated there for centuries. Among these alpine cultivars, we identified nine black-skinned cultivars carrying HapF2 at the BCL*.* This haplotype-sharing pattern is the result of inheritance between close relatives. Indeed, these varieties were shown to belong to a group of genetically closely related cultivars [21–25]. This close genetic relationship between indigenous cultivars from both Valais and Aosta Valley was already proposed based on meticulous ampelographic observations at the beginning of the 20th century [35]. Furthermore, Vouillamoz *et al.* [21] proposed that cv. ‘Rouge du Pays’ could be a progeny from a natural cross between cv. ‘Mayolet’ and cv. ‘Petit Rouge’. However, considering our results and a recent study using single nucleotide polymorphisms (SNP) [23], the pedigree of cv. ‘Rouge du Pays’ proposed by Vouillamoz *et al.* [21] should be revised. In some situations, recent works using SNP analysis do not support the parentage proposed using microsatellite data. One example is the different parentages proposed for ‘Sangiovese’ based on microsatellite markers [36, 37] that were invalidated by two recent studies using SNP analysis [23, 38]. Regarding cv. ‘Rouge du Pays’, we did not find any reliable parental pairs for this cultivar in our database. We conclude, therefore, that at least one of the parents of cv. ‘Rouge du Pays’ is unknown, probably representing an extinct cultivar.

Our results of RNA-Seq analysis showed that, among the nine *MybA*-like sequences from HapF2, only three similar genes were expressed in the skin after veraison: *VvMybA1*, *VvMybA2*, and *VvMybA3*. *VvMybA2_HapF* resembles the functional allele *VvMybA2r*, harboring neither the CA deletion nor the mutation R^44^- > L^44^ [12, 39]. Both *VvMybA1_HapF* and *VvMybA3_HapF* encode a protein of 250 aa in length very similar to the functionally validated protein VvMybA1 identified in other haplotypes [8, 12, 20]. We hypothesize that in the case of HapF, three adjacent *MybA* genes could be involved in activating anthocyanin production in berry skin. Functional assays should be performed in the future to verify this hypothesis. Contrary to what has been observed for other haplotypes in *V. vinifera*, *VvMybA3* in HapF does not contain the 209-bp deletion in the third exon. Interestingly, previous studies and our findings have highlighted this characteristic in an ortholog of *VvMybA3* in haplotype E1 from *V. labrusca* [20, 40].

*VvMybA1_SUB* gene expression was detected in all studied cultivars (cv. ‘Khusaine Belyi’, ‘Otcha Bala’, ‘Cornalin d’Aoste’, ‘Listan Prieto’, and ‘LN33’) except for ‘Sultanina’. Our results for ‘Sultanina’ confirmed the observations made from a previous work [19]. Compared to other expressed *MybA* genes, *VvMybA1_SUB* gene expression occurred at a lower rate in berry skin. A previous study also reported a low transcript level for *VvMybA1_SUB* in cv. ‘Koshu’ compared to the reference allele VvMybA1c in ‘Cabernet Sauvignon’ [41]. The coding part of *VvMybA1_SUB* is identical in HapF and HapF^DEL^; on the protein level, *VvMybA1_SUB* showed 100% similarity (BLOSUM62 > 0) compared to *MYBA* genes that were functionally tested and shown to trigger anthocyanin production [12]. Furthermore, no obvious differences were detected in the promoter region of the gene *VvMyb1_SUB* between HapF and HapF^DEL^. We propose, therefore, the following hypothesis to account for the *VvMybA1_SUB* gene expression and the white-skinned phenotype observed: *VvMybA1_SUB is* functionally active, but its transcript level is not sufficient to activate anthocyanin synthesis via UFGT gene induction. The activation of anthocyanin production by *MybA* transcripts is dose-dependent, based on our results that showed wine anthocyanin content was higher in homozygous genotypes at BCL with two functional haplotypes compared to those with only one (Fig. S15).

The anthocyanin biosynthesis pathway is well conserved in plants, and *MYB* transcription factors have been found to play critical roles in anthocyanin accumulation in different horticultural plants. Various allelic variants of the key regulatory *MYB* transcription factors with their respective phenotypes were identified for different crops [42, 43]. For instance, Liu et al*.* attributed the yellow-skinned fruit (absence of anthocyanin) phenotype of sweet cherry (*Prunus avium*) to a nonfunctional allele due to a large deletion encompassing the *MYB* regulatory gene [44]. For grapevine, HapF^DEL^ described in this work, represents a loss-of-function haplotype responsible for the lack of anthocyanin accumulation in berry skin. BCL showed a high genomic plasticity, and structural variants are common at this locus, as suggested by our results for HapF^DEL^ and the haplotype from *V. labrusca* Grem-4. In grapevines, some nonfunctional or partially functional haplotypes involving large deletions encompassing the BCL have already been reported to be involved in somaclonal variations [45–47]. Interestingly, in our study, we encountered a similar case with cv. ‘Humagne gris’, a bronze-colored somatic mutant of the cv. ‘Cornalin d’Aoste’ [48], which had a chimeric structure [49]. For ‘Humagne gris’, the L1 cell layer had HapF2 at BCL, whereas a deletion occurred in L2 cells eliminating the *MybA* genes of this haplotype. Ancient writings mentioned ‘Oriou gris’, a pale-colored variety, being cultivated in Aosta Valley during the XIX century [50]. ‘Oriou gris’ was most probably a somatic mutant of ‘Petit Rouge’, which was likely derived from it via a mechanism similar to what has been described here for ‘Humagne gris’.

Unlike HapA, the main white berry-skinned haplotype containing *Gret1*, which is present in both table and wine grape cultivars, HapF^DEL^ is found only in table grape varieties and did not migrate to western Europe. Therefore, HapF^DEL^ likely appeared quite recently in the history of grape domestication, probably after the table and wine grape cultivars diverged; this divergence point was estimated to be around 2600 years ago [51]. The most likely evolutive scenario for HapF^DEL^ is that it appeared somewhere in Asia, where it was then selected by humans and disseminated along the ancient Silk Road trade route in the direction of East Asia. Domesticated *V. vinifera* probably reached China and Japan during the last two millennia. To date, the earliest evidence of the presence of *V. vinifera* in China dates back to the second century BCE in the Turpan District in Xinjiang Province [52]. The cv. ‘Khusaine Belyi’ was introduced in China at least as early as 900 CE [53]. Further evidence shows that a cultivated *V. vinifera* accession having HapF^DEL^ naturally hybridized with a descendant of the Chinese wild species *Vitis davidii* to give birth to ‘Koshu’ cultivated at least since the 12th century in Japan [54].

Grapevine domestication and viticulture spreading result from a complex process, and some aspects are still highly debated. For instance, some works suggested a single domestication event in the Near East [55–57], whereas Dong *et al.* [53] proposed two centers of domestication for grapevine, the first one in the Transcaucasus, and the second in the Fertile Crescent (near the Mediterranean). Based on genetic and archeological evidence, researchers generally accept that the Near East region is the primary domestication center(s), after which domesticated grapevine spread to other regions following the main civilizations along human migration routes. Domesticated grapevines spread to Europe around 3000 years ago during the Late Bronze Age [58]. In this study, we found that HapF1 at the BCL had a high frequency (>50%) in the *V. sylvestris* population sampled in the Transcaucasus region and Israel. Furthermore, our results showed that HapF1 was present in an archeological cultivar found in the Middle East dating back to late antiquity (8th century CE). Thus, we can reasonably hypothesize that HapF1 from *vinifera* varieties originated from *sylvestris* populations domesticated in the Near East. It was then disseminated throughout the world as winegrowing spread. We found current *vinifera* varieties bearing F1 from Spain to Uzbekistan (Fig. S16). Our results confirmed the east-to-west gene flow following the spread of domesticated forms of *V. vinifera*.

HapF2, however, followed a different pattern of distribution. It was not detected in the *sylvestris* population but was observed in cultivated varieties but only in a restricted geographical area (in central and north of Italy (Aosta region) and in Switzerland (Valais) at the border with Italy). HapF2 was found in nine closely related alpine cultivars. In addition, only cv. ‘Montepulciano’, which is not directly related to the alpine cultivars, showed the presence of HapF2. This cultivar is one of the most important varieties in central Italy and is the second black-skinned variety planted in terms of surface area in Italy after ‘Sangiovese’. To account for these observations, we can hypothesize that F2 would have originated from a *sylvestris* population in the Italian peninsula; this population is either extinct or has not been captured in the samples we analyzed. Given that cultivated grapes moved into Europe (the origin of Italian viticulture is thought to date back to the Etruscans, around 8^th^ century BCE), it is plausible that western European *vinifera* cultivars encountered local *sylvestris* populations and hybridized with them along migration routes. This putative hybridization could have transferred HapF2 into the cultivated compartment. This putative scenario would be in accordance with a growing body of literature [51, 53, 57, 59, 60] showing that western European *vinifera* cultivars experienced introgression from local *sylvestris*. Our scenario for HapF2 is in agreement with previous observations made by Magris *et al.* [56] that the highest proportion of western *sylvestris* ancestry was found in autochthonous varieties from central and northern Italy.

Overall, our findings offer valuable insights into the genetic mechanisms underlying berry skin coloration in *Vitis vinifera*, enhancing our understanding of grapevine domestication history. We identified a nonfunctional haplotype (HapF^DEL^) responsible for the white-skinned berry phenotype, described here for the first time. By analyzing the geographic distribution of BCL haplotypes, we propose that white-skinned grape cultivars originated independently at least twice during domestication. Furthermore, an improved understanding of the genetic model controlling berry skin color holds significant potential for advancing breeding programs aimed at producing high-quality fruits.

## Materials and methods

### Plant materials

The study was mostly performed using materials originating from two national grapevine repositories: at Agroscope in Pully (VD) in Switzerland and at INRAE in Domaine de Vassal near Montpellier in France. Furthermore, a few accessions were provided from the germplasm repository at JKI Geilweilerhof (Siebeldingen, Germany) and from the Institut Agricole Régional (Aosta, Italy). The genotypes 576P ([‘Rouge du Pays’ × ‘Bronner’] × ‘Voltis’) and ‘Khusaine Belyi’ were selfed to produce homozygous seedlings at the BCL.

To study the genetic component of anthocyanin content in wine, 187 progenies from the cross between cv. ‘Artaban’ and ‘Divico’ were planted (5 stocks per genotype) in 2017 in the field at Pully in Switzerland (46° 30′ 49′ N, 6° 39′ 59′ E). Microvinifications were carried out using a standardized protocol during three vintages, 2020, 2021, and 2022, and wines were analyzed for anthocyanin content using the spectrophotometric method described by Puissant and Leon [61].

### DNA preparation, PCR analysis, and sequencing of *VvMybA1*, *VvMybA2*, and *VvMybA3* alleles

Genomic DNA was isolated from the leaves of field-grown plants or the roots of potted plants in the case of cv. ‘Humagne gris’ using a rapid CTAB procedure [62]. Specific primers were used for the amplification of the different *VvMybA* genes (Table S9). PCR was performed according to the authors’ instructions (Table S9). Amplified DNA was separated on 1.5% agarose gels, stained with ethidium bromide, and directly Sanger sequenced by Fasteris SA (Geneva, Switzerland).

### MinION and Illumina DNA sequencing

DNA was extracted from young leaves using a CTAB procedure [63]. The extract was treated with 1 mg/mL RNase A for 30 min at 37°C. Illumina sequencing of the homozygous genotype (*VvMybA1_SUB / VvMybA1_SUB*) derived from selfing of the breeding line 576P was performed by Novogene (England) using TruSeq DNA library preparation, followed by a NextSeq 6000 sequencing run (2 × 150 bp). This WGS dataset consisted of 71.5 million bp. For long read sequencing, we used the technology of Oxford Nanopore Technologies (ONT), as described by Debonneville *et al.* [63]. Long reads from ONT were *de novo* assembled using Flye v2.8.3 [64] with default parameters. To correct possible assembly/ONT sequencings errors at the BCL, Illumina sequences were mapped using Geneious Prime 2023.2.1 (Biomatters Ltd) and visually inspected.

### Bioinformatic analysis

To identify *MYB* sequences at the BCL, we mapped the conserved part of *VvMybA1* (R2R3 parts of the gene corresponding to exon 1, intron 1, and exon 2) on the investigated contig using Geneious [65] with the highest sensitivity settings. TE annotation at BCL was performed using EDTA [66]. The age of each LTR retrotransposon element was estimated based on the sequence divergence between their LTR pair [67]. Dotplots were used to compare two sets of sequences. We used either D-Genies [68] to compare entire chromosomes/contigs or Gepard v2.0 [69] to compare berry color locus regions. The nucleotide and protein sequences were aligned using MUSCLE V5.1 implemented in Geneious Prime 2023.2.1. Phylogenetics trees were computed using the UPGMA algorithm, Tamura-Nei genetic distance, and bootstrapping with 1000 replicates, as implemented in Geneious Prime 2023.2.1. The consensus tree was rooted with *AtMyb75* as the outgroup. Branches with less than 90% support value were collapsed.

In the analysis of the WGS data, every dataset was mapped using the Geneious mapper against *VvMybA* genes from HapF. To identify HapF, the alignment was visually inspected for the insertion of 33 bp in the second intron of *VvMybA1*; the other *VvMybA* alignments were then examined for confirmation of the presence of HapF. To differentiate the different F subhaplotypes, each alignment was inspected visually, and different criteria were considered: (i) reads spanning the junction of the two tandem copies for detecting the tandem duplication in the region *VvMybA4*; (ii) a SNP located at position 99 of the second intron for *VvMybA1_SUB*; (iii) reads spanning the small insertion of 213 bp in gene *VvMybA4a*, and (iv) reads spanning the junction between RLG transposon and the gene *VvMybA4b*. Identity-by-descent (IBD) analyses were performed on SNP calls retrieved from grapevine whole-genome sequencing data described previously. We followed GATK [70] best practices to call SNPs and subset these to a single SNP per 10 kb window along chromosomes retaining only SNPs with >90% genotyping rate. The filtered VCF file was used in PLINK 2.0 to calculate IBD values with the —make-king option [71].

### Microsatellite analysis

We analyzed five nuclear simple sequence repeats (SSR) markers (VVih54, VVIq52, VViv37, VViv67, and VVMD28) to assess the parentage analysis of cv. ‘Rouge du Pays’. DNA was extracted with the DNeasy Plant Kit (Qiagen) from the young expanded leaves. Amplification was performed on a TProfessional TRIO Thermocycler® (Biometra) and visualized using a 4300 DNA Analyser platform (LI-COR). Allele sizes were determined by comparison with known genotypes of standard cultivars.

### *VvMybA* gene expression analysis in berry skin

Berry samples were taken from field-grown vines. Nine ripe berries of three sun-exposed bunches were collected 6 weeks after veraison. Berry skin was immediately separated from flesh and seeds and directly grounded in liquid nitrogen. Total RNA was extracted from 100 mg tissue using the RNeasy Plant Mini Kit (Qiagen). RT-PCR was performed following the manufacturer’s instructions in one step, starting with 1 μg of total RNA. Each reaction contained the specific primers (Table S9), AMV RT (Promega), RNasin® Ribonuclease Inhibitor (Promega), and GoTaq® Green Master Mix (Promega). The amplified DNAs were separated on 1.5% agarose gel, manually selected according to their sizes, and Sanger sequenced by Fasteris SA (Geneva, Switzerland). For the RNA-Seq experiment, library preparation and transcriptome sequencing were performed at Fasteris (Geneva, Switzerland). Briefly, the stranded mRNA protocol (Poly A selection) was used according to the manufacturer’s instructions for constructing cDNA libraries followed by pair-end sequencing (2 × 150 bp) on an Illumina Novaseq sequencer. Processed reads were mapped against the public transcriptome of *V. vinifera* PN4002412X.v2 [72] using Salmon v1.6.0. [73]. To evaluate the expression of individual alleles of *VvMybA1*, *VvMybA2*, and *VvMybA3,* we initially aligned the nucleotide sequences of all six alleles using MEGA X to identify private SNP (present only in one allele) and then generated a ‘consensus’ *VvMybA* expressed sequence (Table S5).

### QTL analysis of wine anthocyanin content

Genomic DNA extraction from young, expanding leaves was performed using the Qiagen DNeasy 96 Plant Kit, according to the manufacturer’s instructions (Qiagen S.A., Courtaboeuf, France). The 187-progeny population was genotyped using the genotyping-by-sequencing (GBS) method [74]. SNP calling was carried out with GATK [70] after read mapping to the PN40024.v4 reference genome. A genetic map was constructed with Lep-MAP3 [75] and QTL mapping was performed with the R package R/qtl [76]. Sample genotypes were analyzed at the QTL peaks based on the final VCF file used to build the map. The two classes of homozygotes versus heterozygotes at the BCL were sorted based on information from a biallelic SNP at the QTL peak.

## Supplementary Material

Web_Material_uhaf069

## Data Availability

DNA sequencing and RNA-Seq datasets were deposited in the Sequence Read Archive under Bioproject PRJNA1166439.
