# Supplementary material for: A divergent haplotype with a large deletion at the berry color locus causes a white-skinned phenotype in grapevine
Source: Hortic Res. 2025 Mar 6;12(6):uhaf069. doi: 10.1093/hr/uhaf069 (PMC12038235; doi:10.1093/hr/uhaf069)
Supplement: Web_Material_uhaf069 [file web_material_uhaf069.zip › Supplementary_figures_28_1_25.docx]

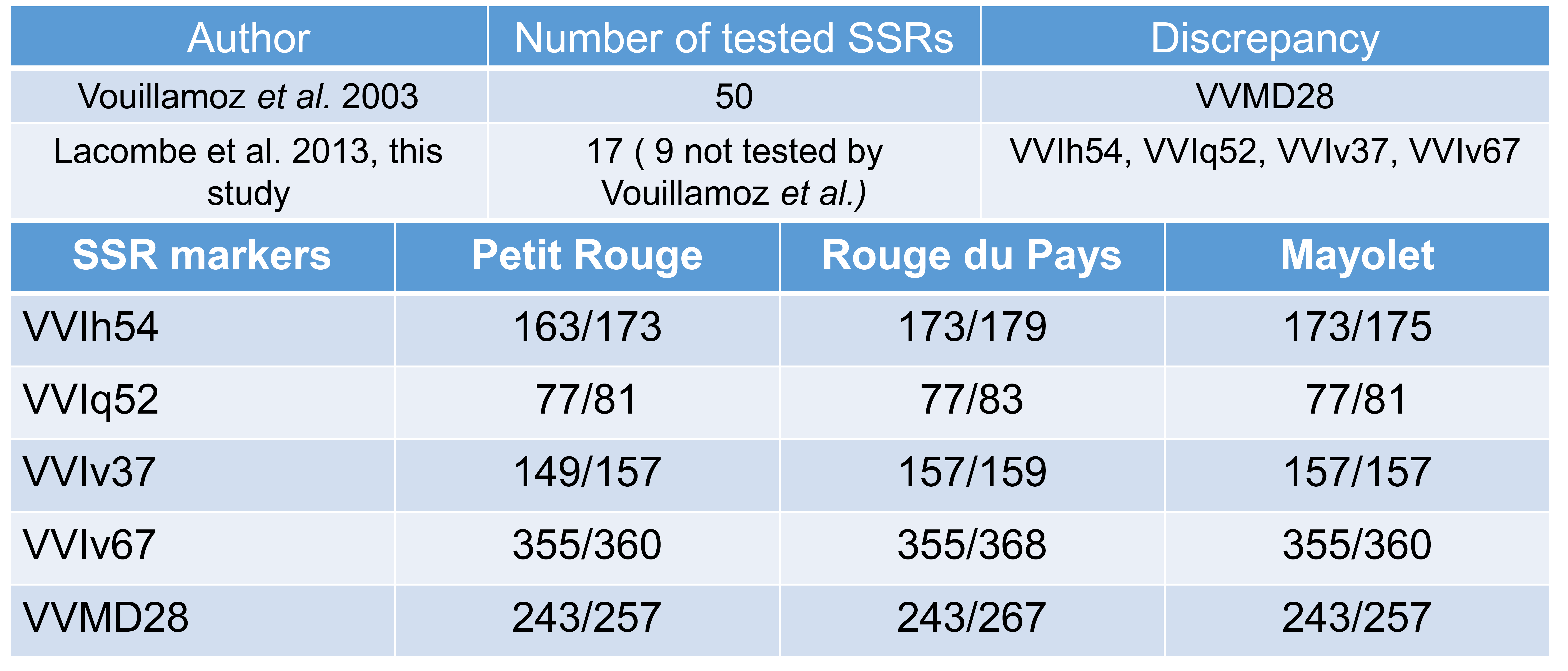


**Figure S1** Summary of SSR marker analysis to test the hypothesis proposed by Vouillamoz et al. (2003) of Rouge du Pays being the result of a natural cross between Mayolet and Petit Rouge (upper panel). In a total of 59 SSR markers analyzed, discrepancies for the suggested pedigree were recorded at 5 loci (lower panel).


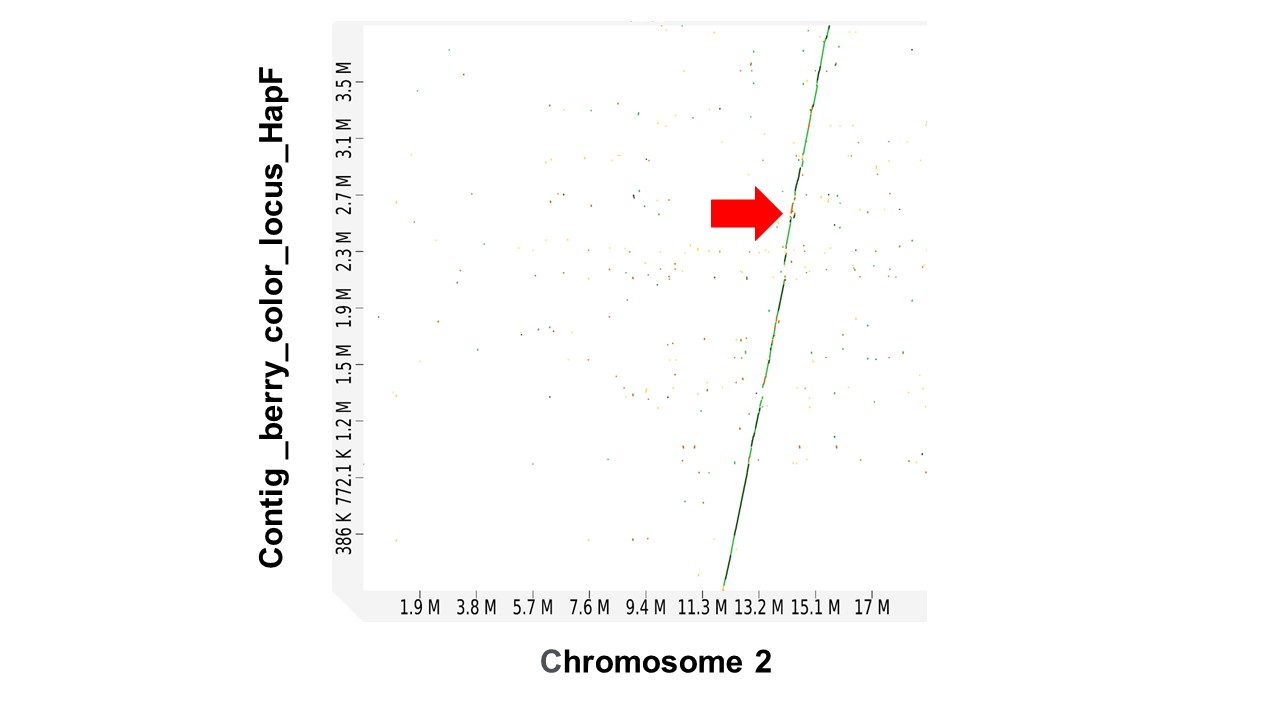


**Figure S2** Dotplot of the contig containing the berry color locus (BCL) of the functional haplotype F (black-skinned berry phenotype) and chromosome 2 from PN40024.v4. The red arrow locates the BCL.


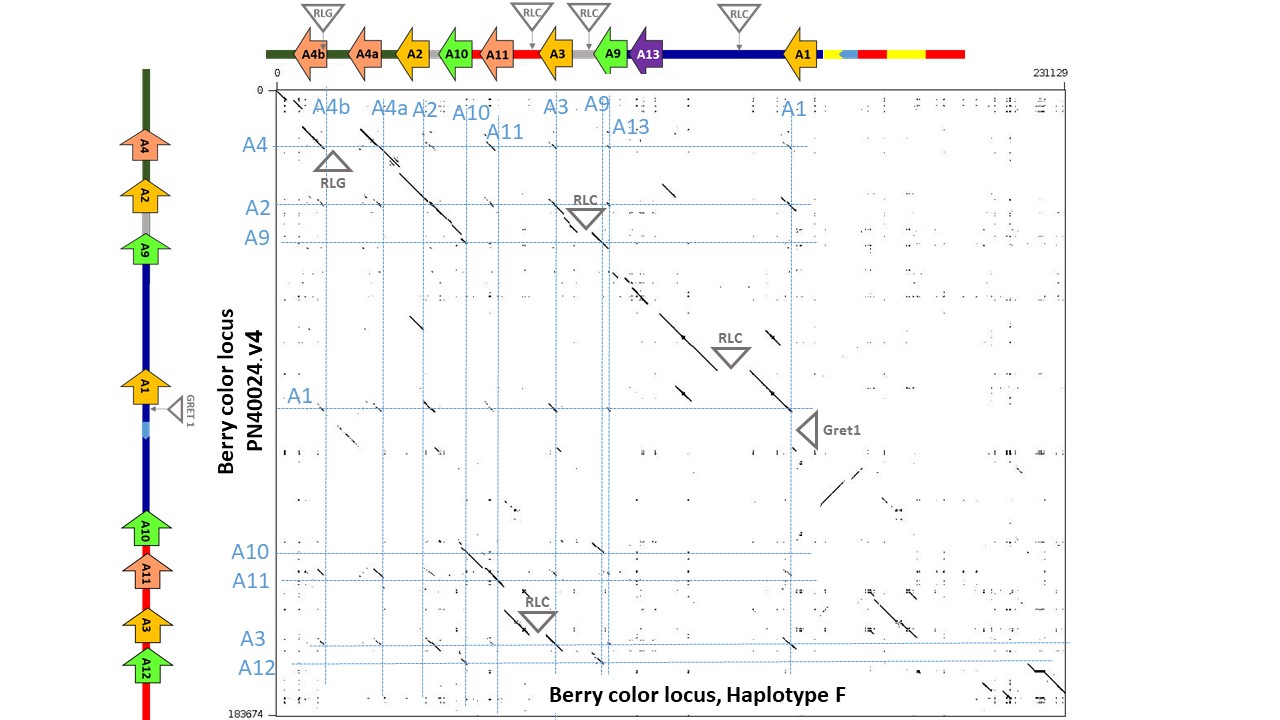


**Figure S3** Dotplot between both loci for berry color in Haplotype A (PN40024.v4) and Haplotype F (Rouge du Pays). Transposable element insertions are shown as gray triangles, two superfamilies were identified: Copia (RLC) and Gypsy (RLG). The blue lines indicate the different *MybA* sequences (denoted here by A, followed by a number).

**
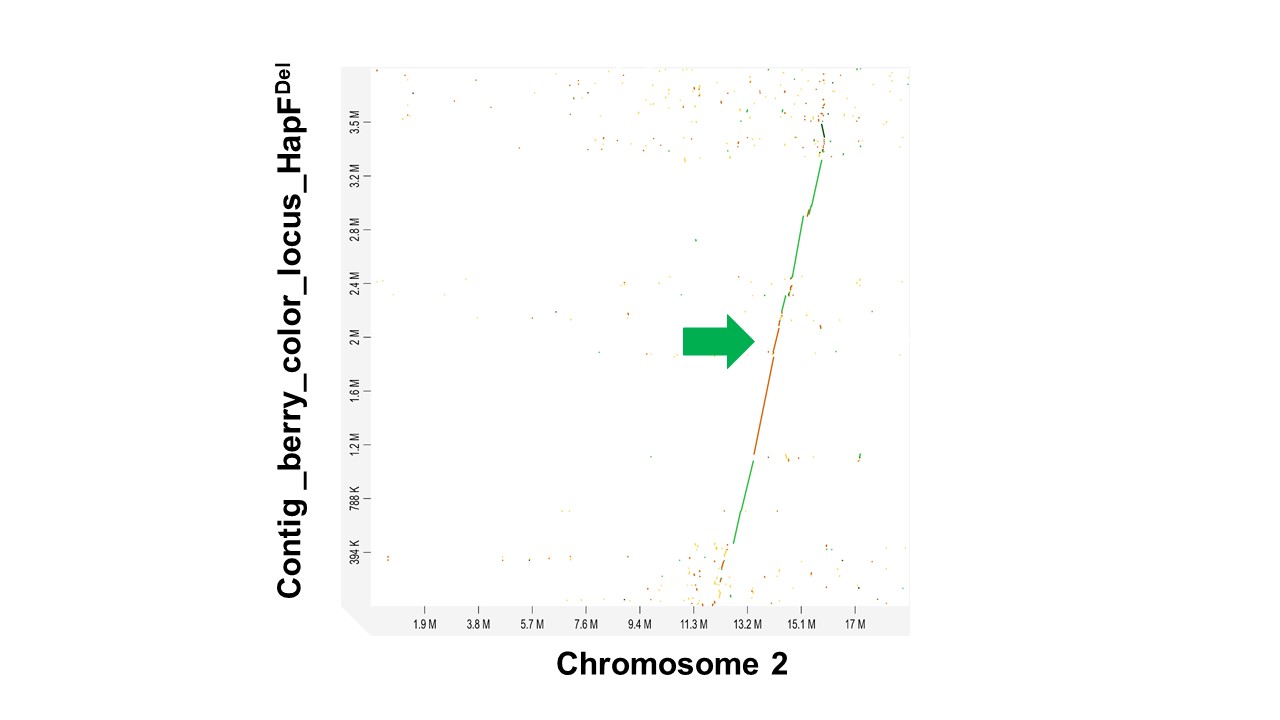
**

**Figure S4** Dotplot between the contig containing the berry color locus (BCL) of the haplotype F^DEL^ (white-skinned berry phenotype) and chromosome 2 from PN40024.v4. The green arrow locates the BCL.


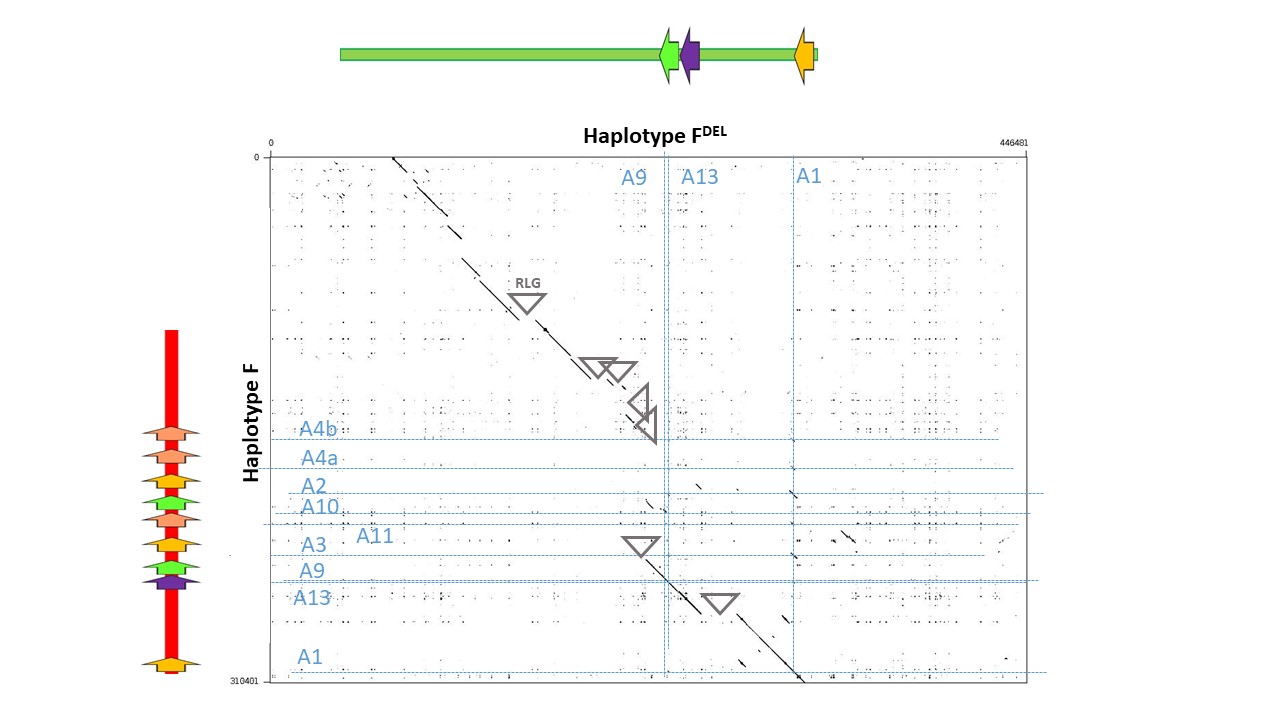


**Figure S5** Dotplot between both loci for berry color in Haplotype F1 and F^DEL^. INDELs are shown as gray triangles. Transposable element insertion is denoted by RLG (Gypsy retrotransposon). Blue lines indicate the different *MybA* sequences (denoted here by A followed by a number).

**
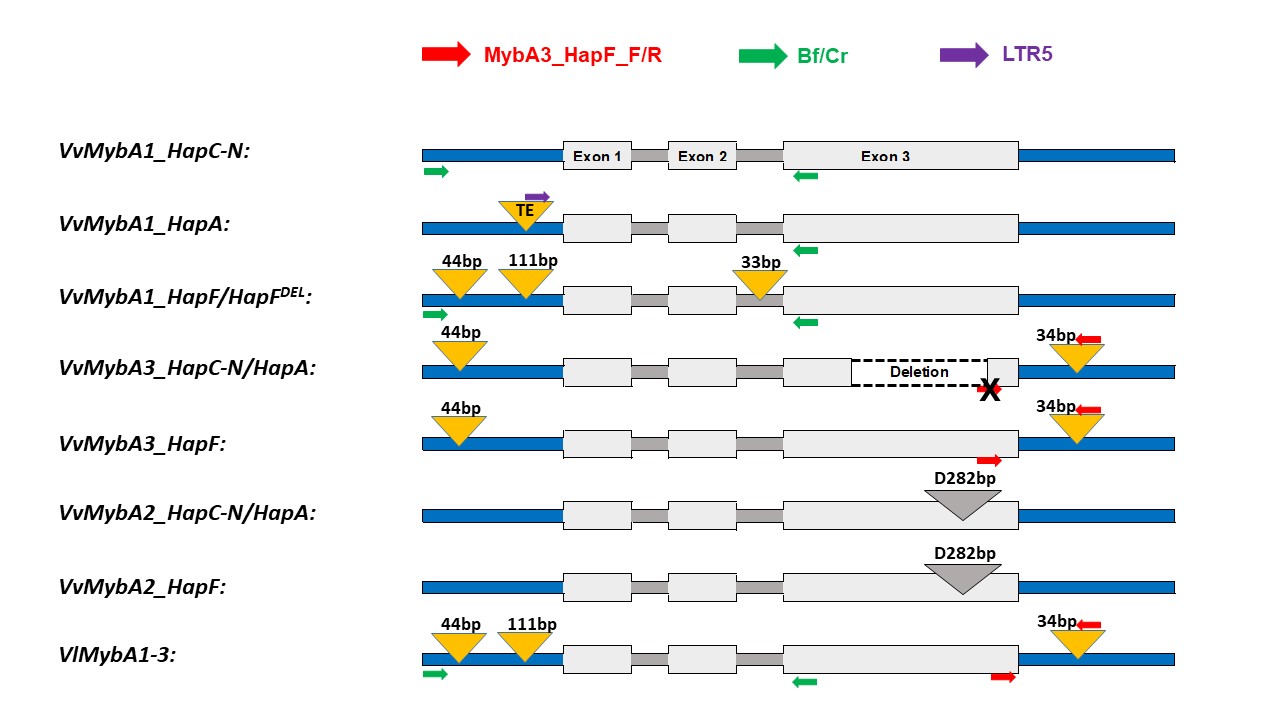
**

**Figure S6** Representation of the different alleles for genes *VvMybA1*, *VvMybA2*, and *VvMybA3* in *Vitis vinifera* compared to one allele identified in *Vitis labrusca* hybrids (*VlMybA1-3*, accession number AB427165). Triangles represent INDELs with a length above in base pairs (bp). The colored arrows represent the different primers used in the PCR for *MybA* genotyping.

**
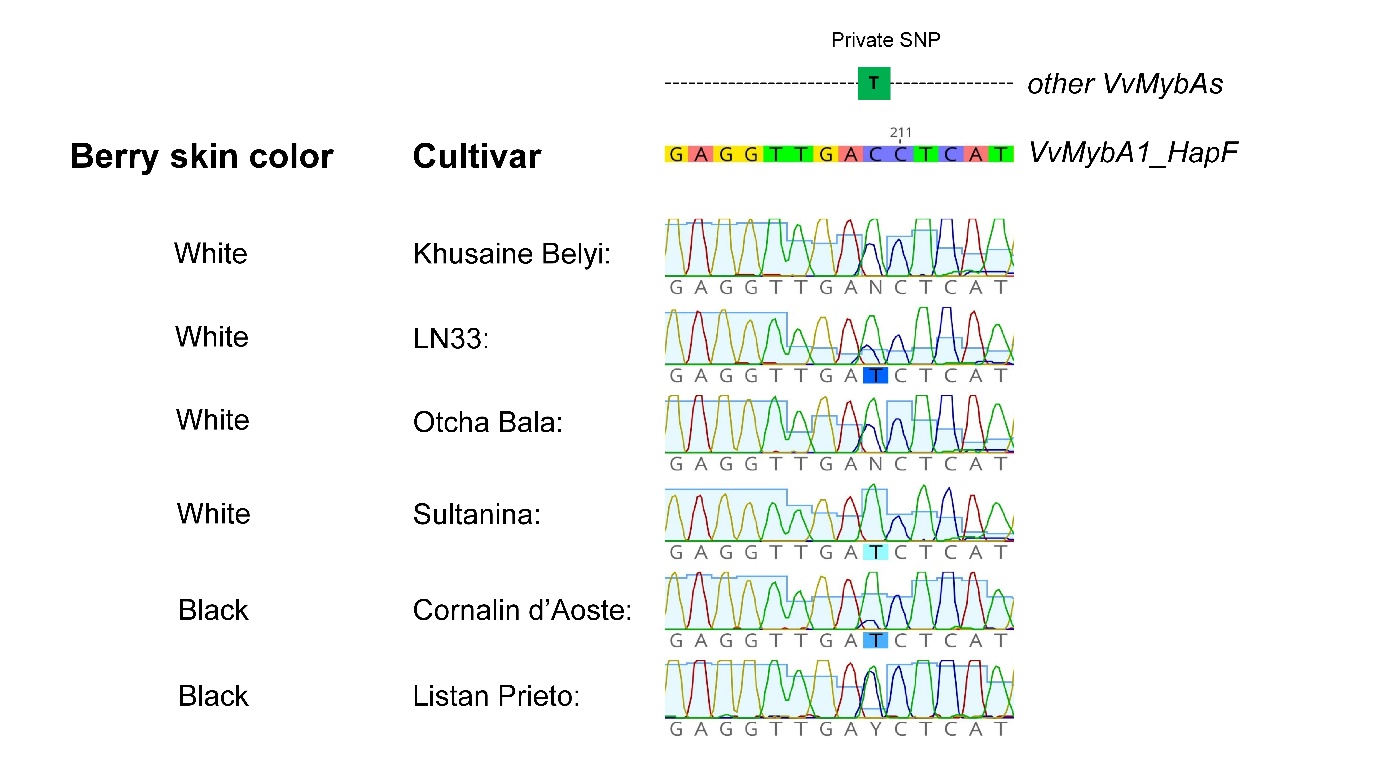
**

**Figure S7** Reverse transcription polymerase chain reaction (RT-PCR, with primers vvMybA1_f and vvMybA1_r) was used to investigate *MybA* gene expression in berry skin after veraison. In the amplified region, cDNA from *VvMybA1_HapF* (=*VvMybA1_SUB*) showed a private SNP (i.d. C is present only in this gene, while other Vv*MybAs* have a T). Chromatogram from DNA sequencing of RT-PCR products showed *VvMybA1_HapF* expression in all investigated cultivars, except Sultanina.

**
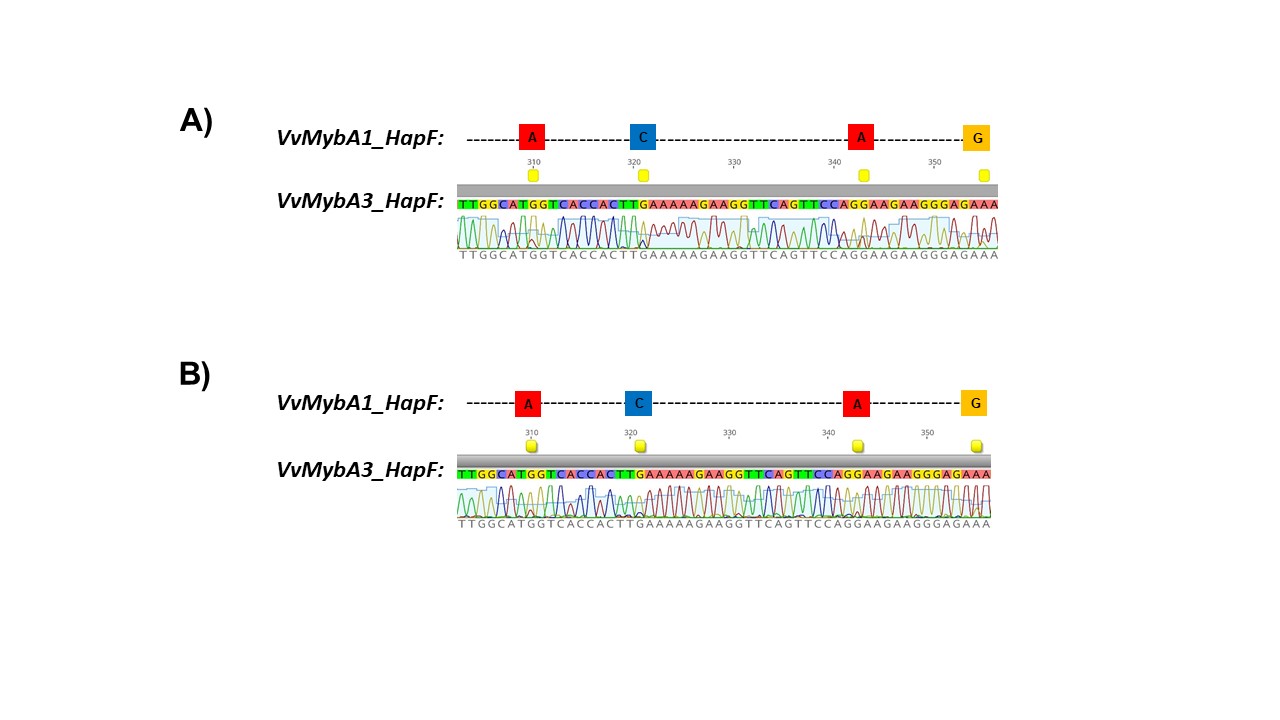
**

**Figure S8** Reverse transcription polymerase chain reaction (RT-PCR, with primers vvMybA1_f and vvMybA1_ Ex3_r) was used to investigate *VvMybA gene expression* in berry skin after veraison. In the amplified region, cDNA from *VvMybA1_HapF* (=*VvMybA1_SUB*) showed several private SNPs (colored in the VvMybA1_HapF sequence). Chromatogram from DNA sequencing of RT-PCR products showed both *VvMybA1_HapF* and *VvMybA3_HapF* expression. However, the peak related to *VvMybA1_HapF* showed a systematically lower signal in the sequencing chromatograms. **A)** Listan Prieto, **B)** Cornalin d’Aoste.




**Figure S9** Comparison of the loci for berry color in haplotype F from *Vitis vinifera* spp. *sativa* cv. Rouge du Pays and two haplotypes identified in *Vitis vinifera* spp. *sylvestris*. The blue lines indicate the different *MybA* sequences (denoted here by A followed by a number). The data for *Vitis vinifera* spp. *sylvestris* were retrieved from Massonnet et al. 2020.

**
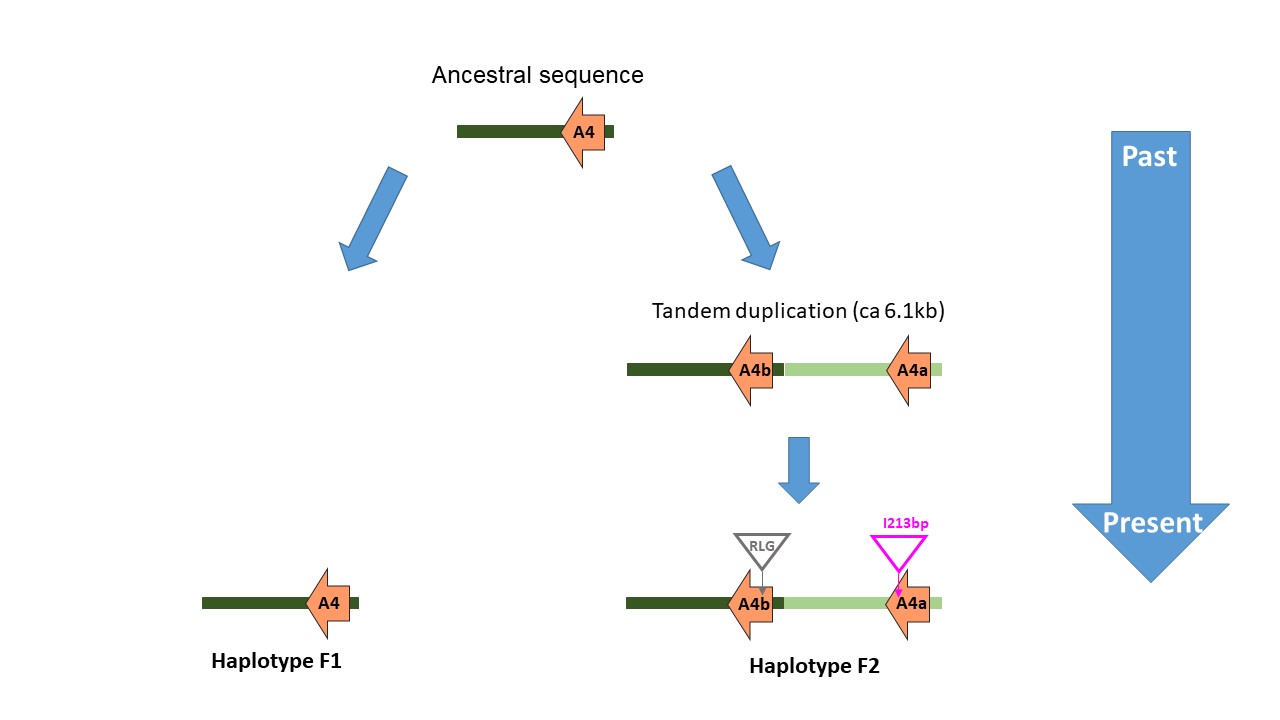
**

**Figure S10** Hypothetical scenario for the evolution of locus *VvMybA4* in haplotype F2. The ancestral haplotype F contained one *VvMybA4* gene; this haplotype split into two around 1.2 million years ago. The subhaplotype F1 is similar to the ancestral one, whereas the second subhaplotype F2 underwent a tandem duplication followed by a transposon insertion (RLG) and a small insertion (I213 bp).


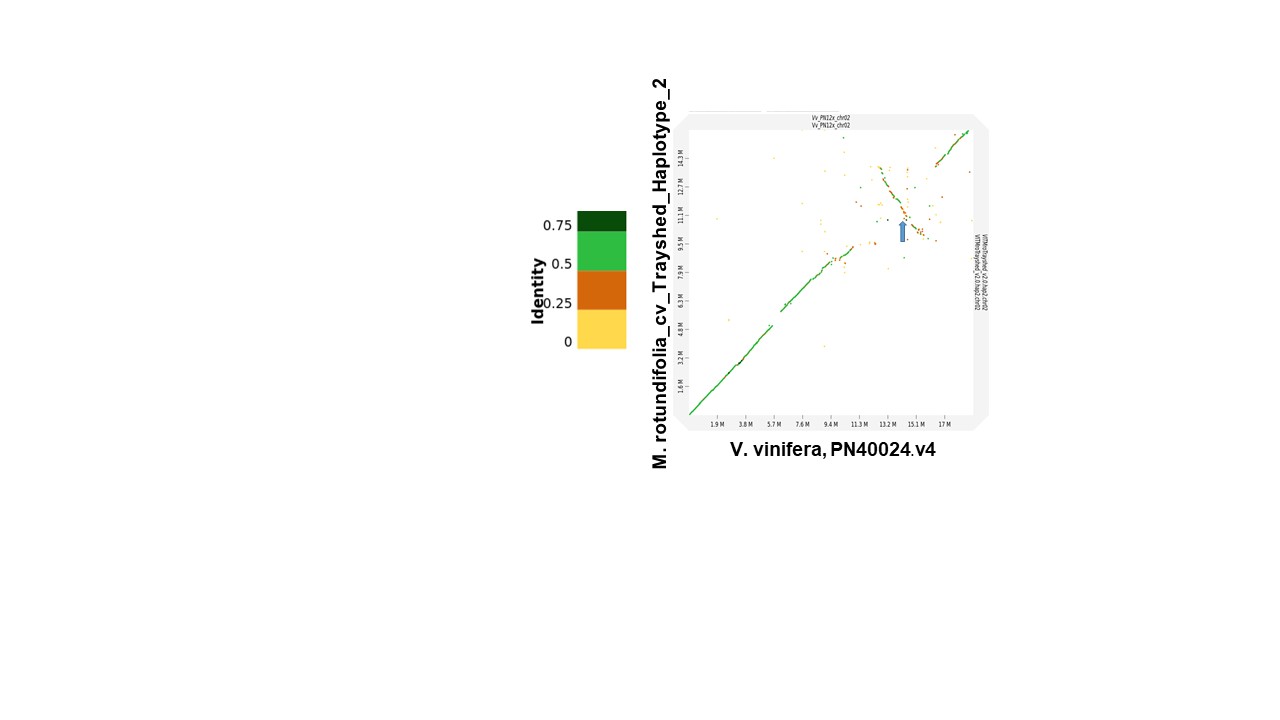


**Figure S11** Dotplot of chromosome 2 from *Muscadinia rotundifolia* (cv. Traysed) and PN40024. The berry color locus from *Vitis vinifera* is indicated by a blue arrow.


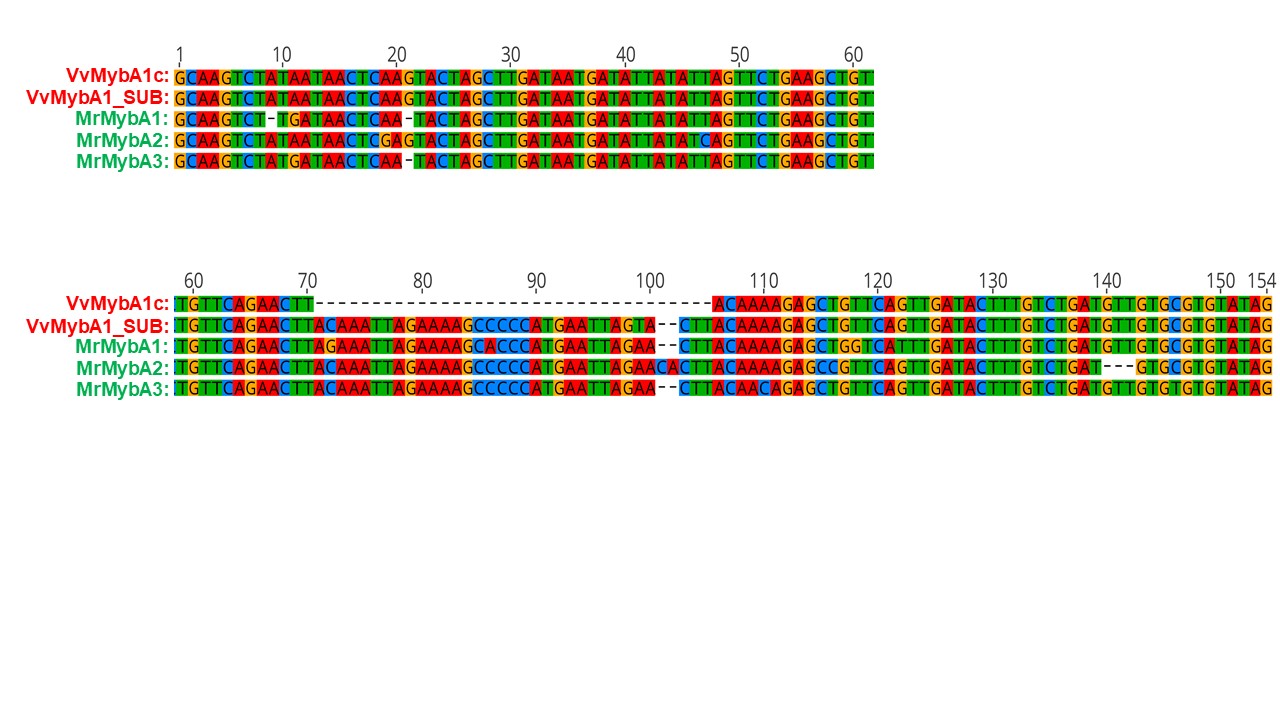


**Figure S12** Alignment of the second intron of several *MybA* genes. Two genes were identified in *Vitis vinifera*: *VvMybA1c* is the functional allele in haplotype C-N, and *VvMybA1_SUB* was observed in haplotype F and F^DEL^. The three genes *MrMybA1*, *MrMybA2*, and *MrMybA3* were observed in *Muscadinia rotundifolia*.

**
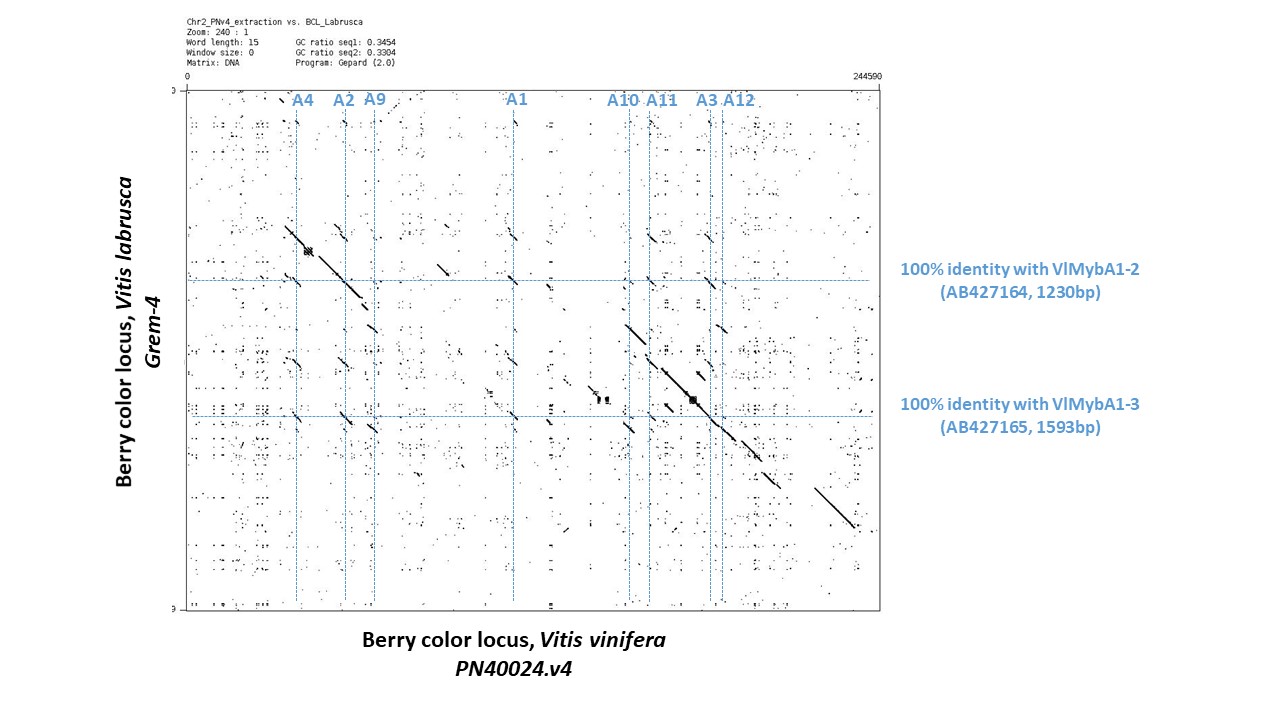
**

**Figure S13** Dotplot of the berry color locus on chromosome 2 from *Vitis labrusca* Grem-4 and PN40024. Blue lines indicate the different *MybA* sequences (denoted here by A followed by a number). The data for *Vitis labrusca* Grem-4 were retrieved from Li and Gschwend (2023).


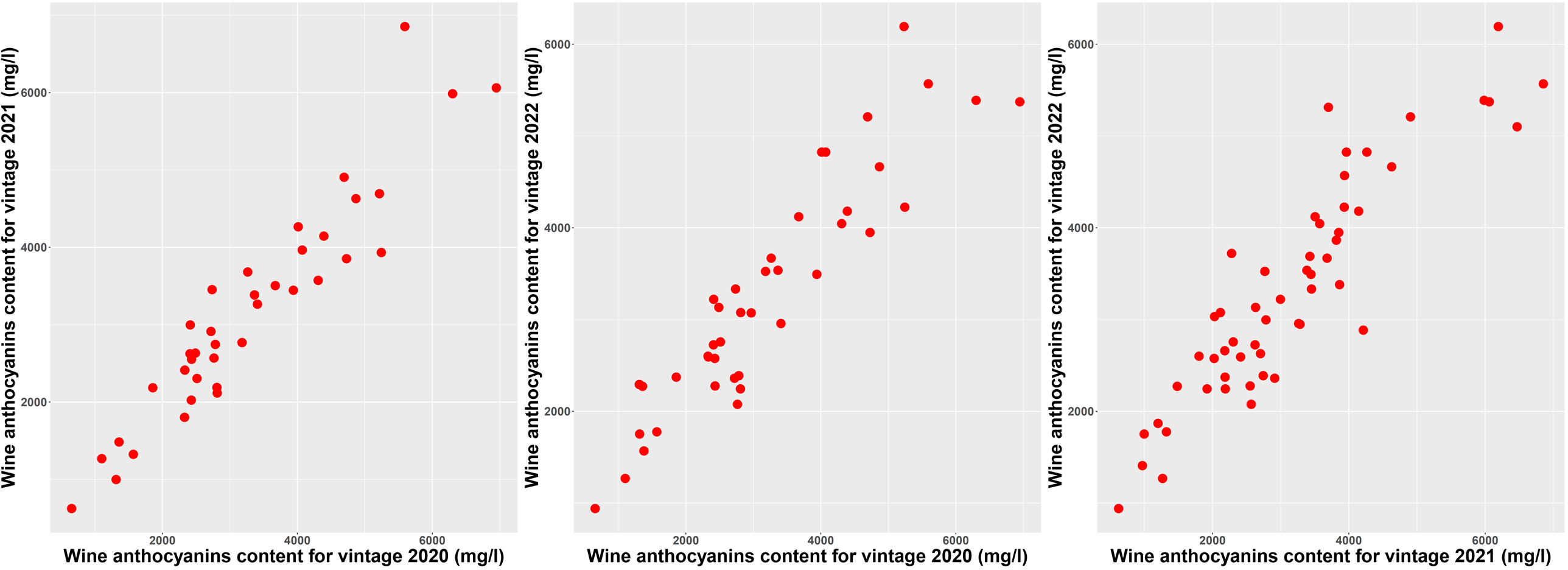


**FigureS14** Wine anthocyanins content between years for given progenies from a cross between cv. Artaban and cv. Divico. 2020 versus 2021 (left panel), 2020 versus 2022 (middle panel), and 2021 versus 2022 (right panel).


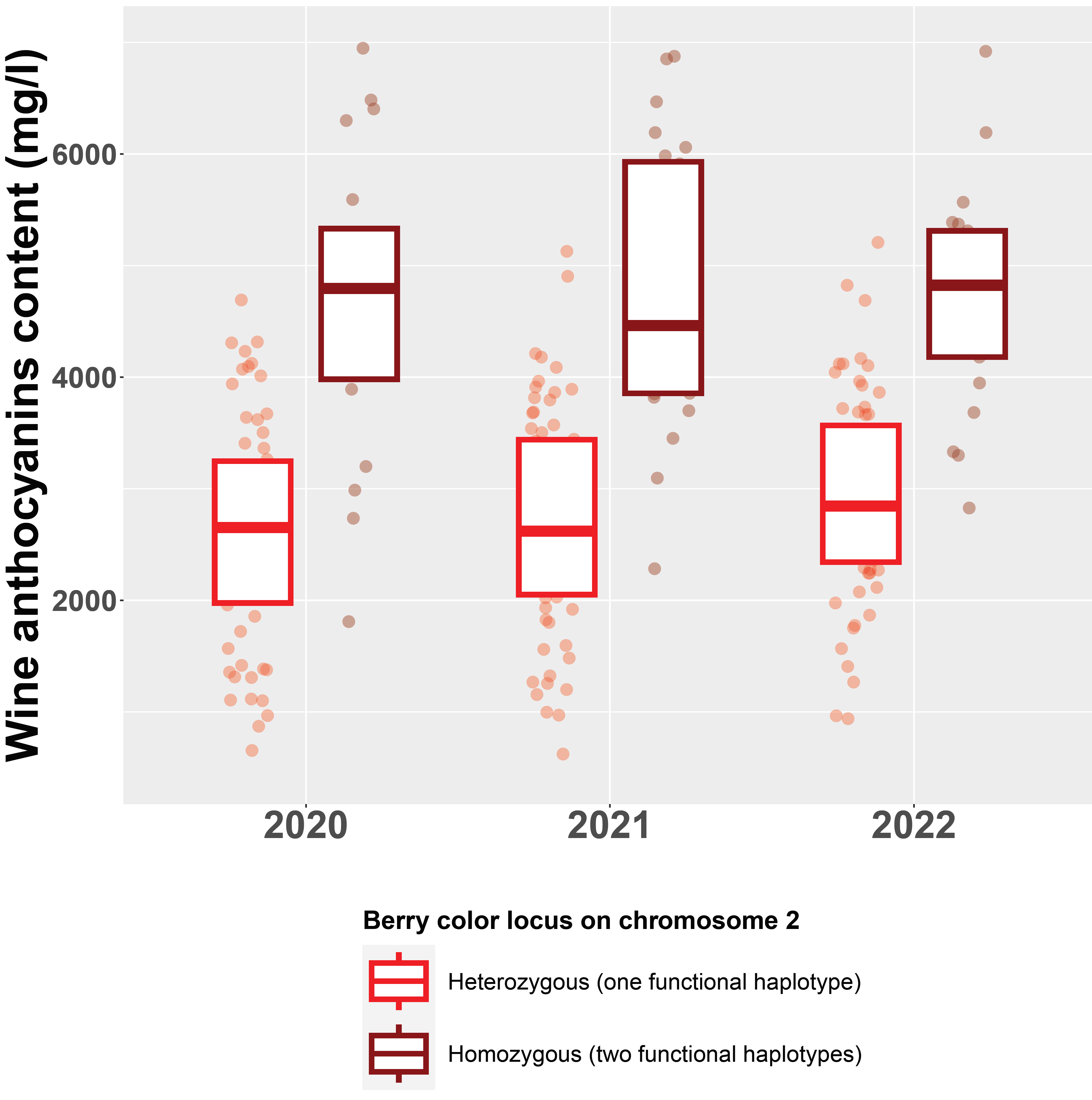


**Figure S15** Relationship between haplotype composition at the berry color locus on chromosome 2 and wine anthocyanin content for three vintages in black-skinned berries progenies from a cross between cv. Artaban and cv. Divico. Heterozygous genotypes possess one functional and one non-functional haplotype (HapA) at the berry color locus. On the contrary, homozygous have two functional haplotypes (2020: heterozygous: n = 62, homozygous: n = 20; 2021: heterozygous: n = 66, homozygous: n = 24, 2022: heterozygous: n= 64, homozygous: n = 21).

**
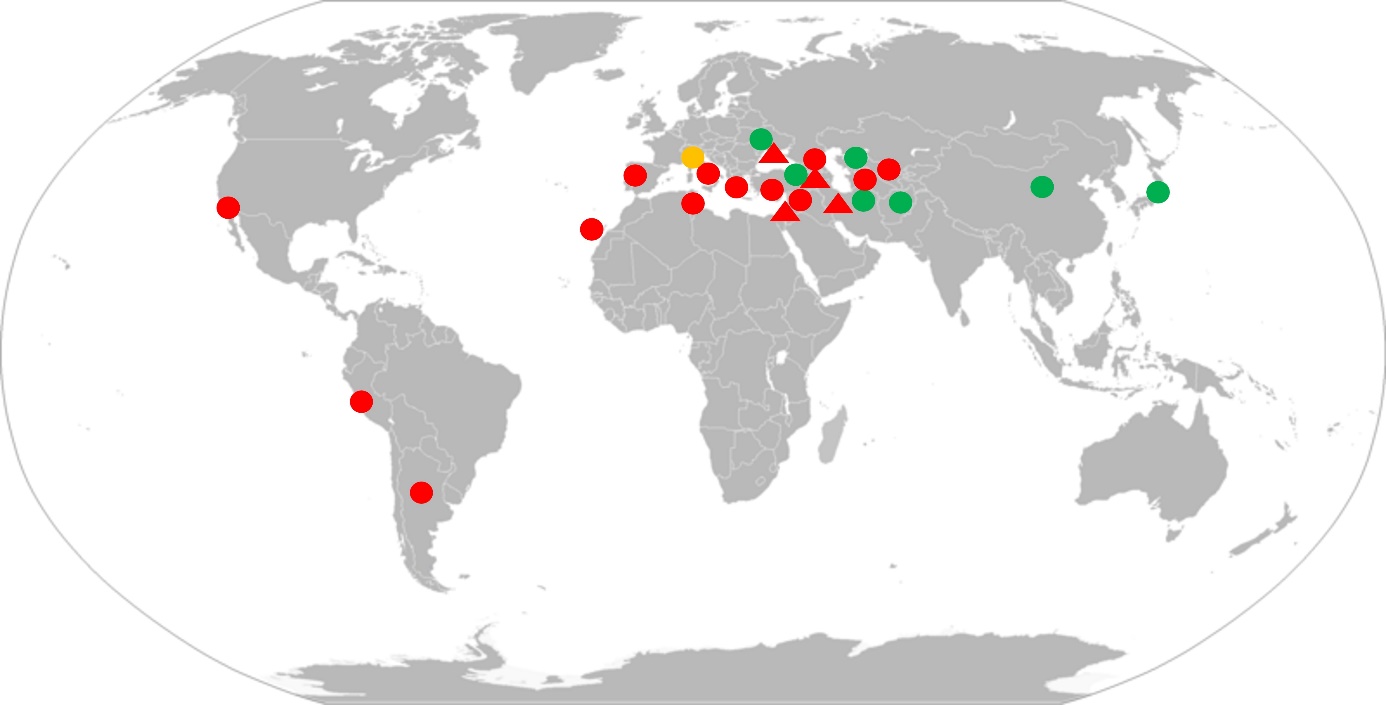
**

**Figure S16** Geographic distribution of haplotype F and its subhaplotypes. Haplotype F is subdivided into three subhaplotypes: the non-functional HapF^DEL^ and two functional ones, HapF1 and HapF2. A circle represents the presence of at least one cultivar with a specific subhaplotype in a region: HapF^DEL^ (green), HapF1 (red) and HapF2 (orange). Circles denote *Vitis vinifera* subsp. *sativa,* while triangles represent *Vitis vinifera* subsp. s*ylvestris.* This information was sourced from Table 3, Supplementary Table S1 and S8.
